# Fatty acid synthesis supports tumor progression through facilitating the activity of TORC1 signaling

**DOI:** 10.1038/s41419-026-08738-6

**Published:** 2026-04-10

**Authors:** Dorottya Károlyi, Sólyom Bálint Bótor, Natali Neuhauser, András Rubics, Janka Szinyákovics, Tibor Kovács, Mária Péter, Gábor Balogh, Fergal O’Farrell, Szabolcs Takáts

**Affiliations:** 1 Department of Anatomy, Cell and Developmental Biology, Eötvös Loránd University, Budapest, Hungary; 2 Doctoral School of Biology, Eötvös Loránd University, Budapest, Hungary; 3 Department of Biochemistry, Eötvös Loránd University, Budapest, Hungary; 4 Department of Genetics, Eötvös Loránd University, Budapest, Hungary; 5 Institute of Biochemistry, HUN-REN Biological Research Centre, Szeged, Hungary; 6 Department of Biological Sciences, University of Bergen, Bergen, Norway; 7 Institute of Genetics, HUN-REN Biological Research Centre, Szeged, Hungary

**Keywords:** Insulin signalling, Macroautophagy, Cancer models, Cancer metabolism

## Abstract

Biosynthesis of lipids and fatty acids (FAs) is essential for the normal functioning of cellular processes, and lipid availability determines the progression of multiple malignant tumor types. To date, the roles of individual steps in lipid biosynthesis during tumor growth and their interaction with intracellular signaling pathways are not well understood. Our study demonstrates that upregulation of de novo FA and lipid synthesis is a conserved characteristic of malignant tumors. In vivo tumor cell-specific silencing of components of the neutral lipid biosynthetic apparatus revealed that loss of several enzymes involved in FA and diacylglycerol synthesis inhibited tumor growth. Specifically, acetyl-CoA carboxylase (ACC), which catalyzes the first step of FA synthesis, drives late-stage tumor growth. FA synthesis perturbation led to inactivation of TORC1 (mechanistic Target of Rapamycin Complex 1)—accompanied by activation of the catabolic process autophagy. Moreover, TORC1 activity cannot be fully restored by hyperactivation of upstream Insulin/PI3K signaling or inhibition of AMP-activated kinase (AMPK) in ACC-deficient tumor cells, but supplementation with ectopic oleic acid can partially increase TORC1 activity and tumor progression. In addition to their metabolic value, the role of FAs in promoting TORC1 gives us new insight into cancer cell dependence on de novo FA synthesis.

## Introduction

Cancer-associated reprogramming of metabolism in cancer cells and the non-transformed tissues of the host is critical for tumor progression [1, 2]. In addition to glucose and amino acids, sufficient lipid availability is also critical for the progression of multiple tumor types. The lipid demand of tumor cells can be fulfilled both from external sources—food intake, or lipolysis mediated release from host tissues—, or de novo synthesis. In contrast to most cell types (except adipocytes and hepatocytes) that obtain lipids from external sources, increased fatty acid/FA and lipid synthetic activity is characteristic of multiple tumor types [1, 3], and targeting lipid biosynthesis is considered as a potentially effective anti-cancer strategy [4, 5].

Glycerolipids (GLs) are the most prevalent lipids composed of a glycerol or a glycerol-3-phosphate backbone esterified with two or three FAs (diacylglycerols/DGs, triacylglycerols/TGs) or one or two FAs and a polar headgroup (glycerophospholipids/GPLs). GLs can serve as storage lipids (TGs), membrane components (GPLs), and signaling molecules (DGs, phosphoinositides) [1]. The synthesis of FAs and GLs begins with Acetyl-CoA that is converted to malonyl-CoA by Acetyl-CoA Carboxylase(ACC) enzyme, and malonyl-CoA is used by fatty acid synthase (FASN) to build an elongated FA chain [1, 6]. Although several steps of FA and GL synthesis have been recognized as critical for tumor progression, how cancer cells sense FA and lipid availability and how this influences growth signaling is poorly understood.

Insulin/PI3K/TORC1 signaling is the central regulator of FA synthesis by promoting the expression of ACC or FASN [7, 8]. TORC1 is active under nutrient-rich conditions and promotes anabolic processes and cell growth [9, 10]. TORC1 senses nutritional status (like amino acid availability) [11, 12] and the energetic state of the cells, by integrating input from AMP-activated kinase (AMPK) which is activated upon high AMP/ATP ratio and attenuates anabolism through inhibitory phosphorylation of TORC1 [13] and ACC [14]. Elevated expression of ACC and Insulin/PI3K/TORC1 activity is characteristic of multiple tumor types [1, 8, 15]. Genetic or pharmacological inhibition of ACC efficiently attenuates the progression of cultured and xenografted cancer cells by increasing oxidative stress and apoptosis [16–19] or decreasing proliferation [20]. As Insulin/PI3K/TORC1 signaling stimulates cell proliferation and inhibits apoptosis [15], it raises the possibility that loss of ACC may also affect TORC1 activity in these tumors through a yet uncharacterized mechanism.

Recently, *Drosophila* became a popular model organism for studying how alterations of signaling and metabolic pathways in tumors and host tissues affect tumor progression in a living organism [21]. The progression of a well-established *Drosophila* carcinoma model is proven to be dependent on the import of sugars and amino acids that are released from host tissues [22]. However, whether these tumors also require lipid import or instead they rely more on de novo synthesis is not known. Hence, in our current research we use the same *Drosophila* tumor model to carry out cancer cell-specific inhibition of distinct steps in FA and lipid synthesis, to uncover the cell-autonomous role of these processes in tumor progression and to reveal how defective FA synthesis affects Insulin/PI3K/TORC1 signaling in vivo.

## Results

### Cancer cell-specific FA synthesis is required for lipid droplet formation and tumor growth

**Fig. 1:**
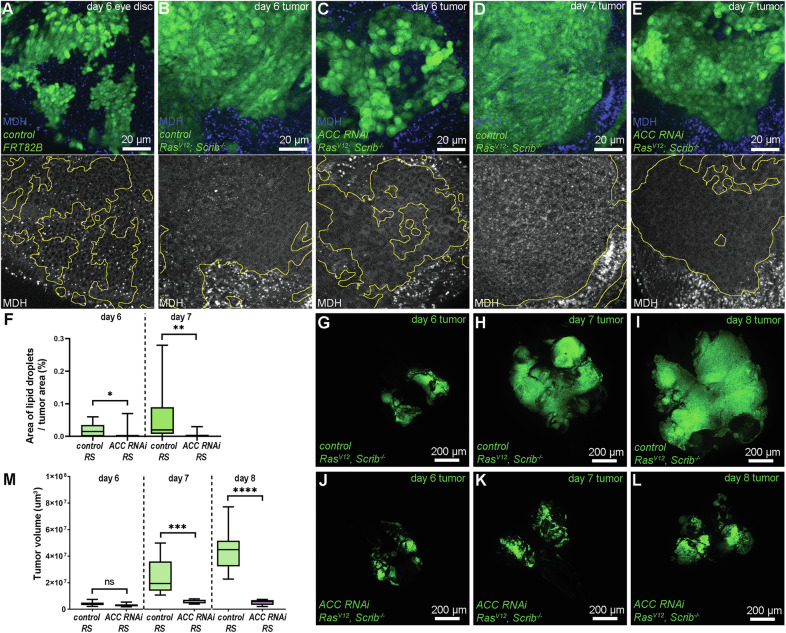
Activation of de novo FA synthesis and LD formation supports the growth of RS tumors. **A** LDs are evenly distributed in control eye-antennal discs, and their formation is not affected by the generation of control MARCM clones (GFP + ). **B**, **C** In early-staged (day 6), control RS tumors (**B**) LDs persist in non-transformed microenvironment (GFP-) but mostly disappear from cancer cells (GFP + ), and this LD depletion is even more prominent upon cancer cell-specific loss of ACC (**C**). **D**, **E** In mid-late stage (day 7), LDs can be detected in control RS tumor cells (GFP + ), however their amount is still lower than in the microenvironment (GFP-) (**D**). In contrast, LDs are still undetectable in the same staged ACC-deficient cancer cells (**E**). **F** Quantification of data presented in (**B**–**E**). In total, 10–15 tumors/genotypes were analyzed, *n* = 10 (**B**), 15 (**C**), 10 (**D**), 13 (**E**). Mann–Whitney tests, *P < 0.05; **P < 0.01. **G**–**L** Representative images about early (day 6), mid-late (day 7), and late-staged (day 8) control (**G**–**I**) and ACC-deficient (**J**–**L**) RS tumors. **M** Quantification of data presented on (**H**–**M**). In total, 8–11 tumors/genotypes were analyzed, *n* = 10 (**G**), 10 (**H**), 9 (**I**), 11 (**J**), 9 (**K**), 8 (**L**). Welch’s *T* test, ns: non-significant; ***P < 0,001; ****P < 0,0001. GFP+ areas are encircled with yellow in grayscale panels of (**A**–**E**).

To characterize the importance of lipid synthesis in *Drosophila* tumor progression, we induced malignant tumors in the rapidly proliferating larval eye-antennal disc epithelium by generating GFP-positive cell clones that overexpress the oncogenic *Ras*^*V12*^ allele and become homozygous mutant for the cell polarity gene *Scribble/Scrib* (hereafter, these are referred as *Ras*^*V12*^*, Scrib*^*−/−*^/RS tumors) [21]. Lipid droplets (LDs), the main lipid storage organelles, are present in every healthy imaginal disc cell (Fig. 1A). To assay malignancy-associated reprogramming of lipid metabolism, we generated control and FA synthesis-deficient RS tumors—by silencing ACC specifically in tumor cells—and stained them with the lipophilic dye monodansylpentane (MDH). Interestingly, in early stage (day 6 after egg laying) the number of LDs is very low in the control tumor tissue but not in the microenvironment (Fig. 1B, F). Expectedly, LDs were completely depleted in ACC-deficient cancer cells (Fig. 1C, F), indicating that early-staged cancer cells may utilize all intracellular storage lipids. In contrast, in mid-late stage (day 7), we observed the reappearance of LDs in control RS tumors (Fig. 1D, F), however LDs were still lacking in ACC-deficient ones (Fig. 1E, F), indicating that there is an ACC-dependent shift in FA and lipid synthesis during the transition from the early to mid-late stages. We measured the size of control and ACC-silenced RS tumors at early (day 6), mid-late (day 7), and late (day 8) stages. Although the loss of ACC decreased tumor size at any stage, this was only significant in mid-late and late stages (Fig. 1G–M). The tumor tissue/microenvironment ratios were also similar on day 6 but not in later-staged control RS and ACC RNAi RS tumors (Supplementary Fig. S1). Hence, ACC-mediated FA synthesis is required mostly for supporting the accelerated progression of RS tumors in later stages, but it is not yet limiting during the growth of early tumor tissues.

### Cancer cell autonomous FA and DG synthesis is critical for tumor progression

**Fig. 2:**
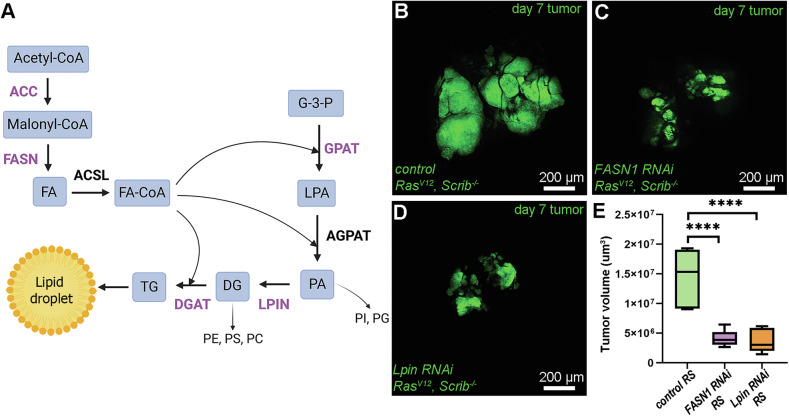
FA and DG synthesis is critical for the progression of RS tumors. **A** Schematic figure about the biosynthetic pathway of neutral lipids, by representing lipid classes and organic intermediate products (placed in rectangles) involved in the neutral pathway and the major families of lipid biosynthetic enzymes (placed above the arrows). The enzyme families that were tested in the small-scale screen are represented by purple letters. **B**–**D** Representative images at day 7 control (**B**) and FASN1 (**C**) or Lpin (**D**) deficient RS tumors from the RNAi screen. **E** Quantification of respective tumor size data represented on (**B**–**D**). In total, 7–8 tumor/genotypes were analyzed, *n* = 7 (**B**), 8 (**C**), 7 (**D**). One-way ANOVA, ****P < 0,0001. FA fatty acid, G-3-P glycerol-3-phosphate, LPA lysophosphatidic acid, PA phosphatidic acid, DG diacylglycerol, TG triacylglycerol, PI phosphatidylinositol, PG phosphatidylglycerol, PE phosphatidylethanolamine, PC phosphatidylcholine, PS phosphatidylserine, ACC acetyl-CoA-carboxylase, FASN1 fatty acid synthase 1, ACSL acyl-CoA-synthase, GPAT glycerol-3-phosphate acyltransferase, AGPAT 1-acylglycerol-3-phosphate-O-acyltransferase, LPIN Lipin, DGAT DG acyltransferase.

Since LDs are mostly composed of neutral lipids (Fig. 2A), we carried out a small-scale RNAi screen by knocking down various enzymes required for neutral lipid synthesis, specifically in the RS cancer cells, and assayed their effect on tumor size. Similarly to ACC, silencing FASN1 also caused a dramatic decrease in tumor size (Fig. 2B, C, E), confirming that RS tumors are highly dependent on de novo FA synthesis. We also silenced several enzymes involved in later stages of lipid synthesis and found that loss of Lpin phosphatidic acid phosphatase (generates DG from phosphatidic acid/PA), strongly inhibited tumor growth (Fig. 2B, D, E). In contrast, silencing genes encoding glycerol-3-phosphate acyltransferase (GPAT) enzymes *mino* or *Gpat4* (Supplementary Fig. S2A, B), or the DG acyltransferase (DGAT) *mdy* did not perturb tumor progression (Supplementary Fig. S2C), while the efficiency of these RNAis was verified by qPCR analysis, and (for mdy) decreased lipid staining in fat body cells of early L3 larvae (Supplementary Fig. S2D–G).

### De novo FA synthesis protects tumor cells from apoptosis

**Fig. 3:**
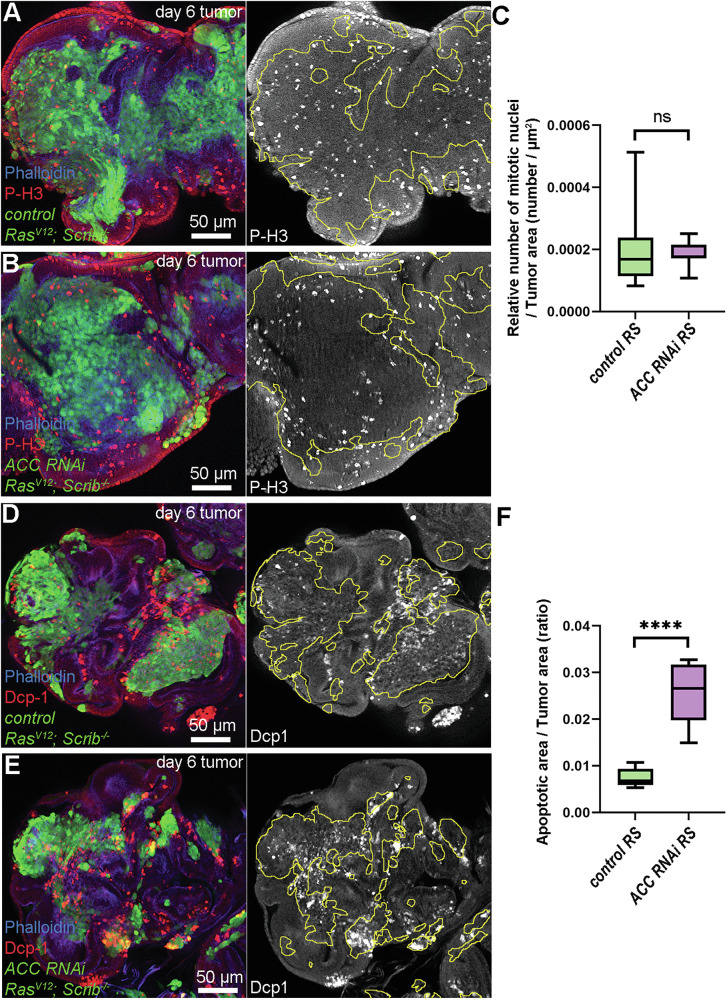
Loss of ACC promotes cancer cells’ death but does not affect their proliferation. **A**, **B** Comparison of control (**A**) and ACC-deficient (**B**) tumor (GFP + ) in the relative number of mitotically active cells (positive for anti-P-H3 staining) compared with microenvironmental cells (GFP−). **C** Quantification of data presented in (**A**, **B**). In total, 9–10 tumors/genotypes were analyzed, *n* = 10 (**A**), 9 (**B**). Mann–Whitney test, ns: non-significant. **D**, **E** Immunolabeling of active effector caspase Dcp-1 for comparison of ACC-deficient tumor tissue (GFP + ) or control to microenvironment (GFP−). **F** Quantification of data presented in (**D**, **E**). Six tumors/genotypes were analyzed, *n* = 6 (**D**, **E**). Welch’s *T* test, ****P < 0,0001. GFP+ tumor areas are encircled with yellow in grayscale panels of (**A**, **B**, **D**, **E**).

RS tumors characteristically display rapid proliferation and large apoptotic areas located mostly in the surrounding wild-type microenvironment [23]. To assess the intensity of these two processes, we carried out immunolabeling on day 6 tumors. By staining nuclei of proliferating cells with Phospho-Histone H3 (P-H3), we did not observe any difference between cancer cell clones of control or ACC-deficient RS tumors in the frequency of mitotically active cells (Fig. 3A–C). In contrast, ACC-deficient cancer cell clones overlapped with apoptotic marker cleaved Death Caspase-1/Dcp-1 staining to a significantly larger extent than controls did (Fig. 3D–F). This indicates that elevated FA synthesis has a cytoprotective effect that helps cancer cells to evade apoptosis.

### Tumor-specific loss of ACC reduces TORC1 activity

**Fig. 4:**
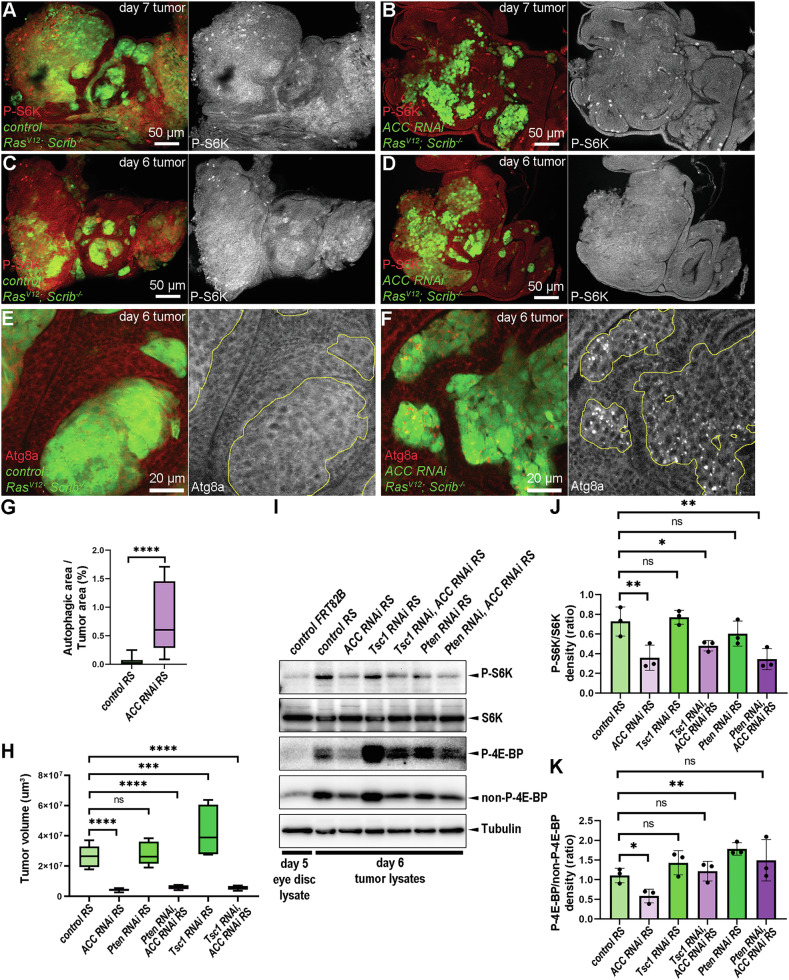
Tumor cell-specific loss of ACC inactivates TORC1 and makes it non-receptive for incoming signals of the Insulin/PI3K pathway. **A**–**D** Anti-P-S6K immunolabelling to investigate TORC1 activity in early (day 6) and mid-late-staged (day 7) control (**A**, **C**) and ACC-deficient (**B**, **D**) tumors (GFP + ) and their microenvironment (GFP-). **E**, **F** Immunolabelling with anti-Atg8a reveals the number and distribution of autophagic structures in control (**E**) and ACC RNAi (**F**) RS tumor cell clones (GFP + ). **G** Quantification of data presented in (**E**, **F**). 10-13 tumors/genotypes were analyzed, *n* = 13 (**E**), 10 (**F**). Mann–Whitney test, ****P < 0,0001. **H** Tumor size analysis on day 8 tumors reveals how cancer cell-specific loss of Pten and Tsc1 affects the growth potential of control and ACC-deficient RS tumors. In total, 6–10 tumors/genotypes were analyzed, *n* = 8 (control RS), 10 (ACC RNAi RS), 8 (Pten RNAi RS), 6 (Pten RNAi, ACC RNAi RS), 7 (Tsc1 RNAi RS), 9 (Tsc1 RNAi, ACC RNAi RS). One-way ANOVA, ns: non-significant; ***P < 0,001; ****P < 0,0001. **I** Western blotting on day 6 tumors to reveal the effect of loss of Pten or Tsc1 on the P-S6K/S6K and P-4E-BP/non-P-4E-BP ratios in control and ACC-deficient tumors. Tubulin represents a loading control. **J**, **K** Densitometry analysis of P-S6K/S6K (**J**) and P-4EBP/non-P-4EBP (**K**) ratios based on the experiment in (**I**). Three parallel western blot experiments were analyzed (*n* = 3/genotype). Repeated measures ANOVA, ns: non-significant *P < 0,05, **P < 0,01.

Considering the weaker growth potential and survival of ACC-deficient RS cells, we asked whether loss of ACC may perturb TORC1 activity. Active TORC1 not only promotes translation by phosphorylating S6-Kinase (S6K) and elongation initiation factor 4E binding protein (4E-BP), but also inhibits the lysosomal catabolic process, autophagy [10, 24]. Immunolabeling with anti-P-S6K revealed that cancer cells in early and mid-late-stage control RS tumors show elevated TORC1 activity compared to the microenvironment (Fig. 4A, C). This was not observed in ACC-deficient cancer cells (Fig. 4B, D), indicating the activity of TORC1 is comparatively reduced. Immunolabeling of autophagic marker Atg8a further supported this conclusion. While Atg8a-positive structures were rare in control tumors (Fig. 4E, G), ACC-deficient cancer cells showed the accumulation of Atg8a-positive autophagosomes (Fig. 4F, G).

We supposed the growth of these tumors might be partially restored by reactivating TORC1 through ectopic induction of Insulin/PI3K pathway. Hence, we generated tumors that simultaneously silenced ACC and known negative regulators of Insulin/PI3K pathway, Phosphatase and Tensin Homolog (Pten) and Tuberous Sclerosis 1 (Tsc1) with RNAi transgenes that were verified by qPCR and an efficient inhibitory effect on starvation-induced autophagy in fat cells of early L3 larvae [24] (Supplementary Fig S3A–C). Reducing levels of Pten and Tsc1 caused a mild overgrowth in both control and ACC-deficient RS tumor conditions at day 8 (Fig. 4H and Supplementary Fig. S3D–I) but could not restore ACC RNAi RS tumors to sizes comparable to RS tumors under any manipulation, indicating that enhancing Insulin/PI3K signaling could not override the ACC deficiency-associated low TORC1 activity.

Western blots assaying phosphorylated/total S6K ratios in day 6 tumor lysates demonstrated elevated TORC1 activity in RS tumors compared to control eye discs and ACC RNAi tumors and confirmed that silencing of Pten or Tsc1 in ACC-deficient tumors had negligible effects on TORC1 activity, indicating that activation of Insulin/PI3K signaling was inefficient in stimulating TORC1 in ACC-deficient samples (Fig. 4I, J). This result suggests that the low growth potential of ACC-deficient RS tumors is due to constantly blunted TORC1 activity. Although decreased P-4E-BP/non-P-4E-BP ratios also indicated lower TORC1 activity in ACC-deficient tumors compared to controls, we also observed that Pten and Tsc1 RNAis caused an increase in the P-4E-BP/non-P-4E-BP ratios and the cumulative levels of non-P- and P-4E-BP proteins in both RS and ACC RNAi RS backgrounds. While loss of ACC caused a strong decrease of 4E-BP protein levels compared to RS controls under all parallel genetic conditions (Fig. 4I, K). Hence, alterations in the P4E-BP/non-P-4E-BP ratios can be partially due to ACC and Insulin signaling-dependent changes in 4E-BP expression. Importantly, the increased levels of 4E-BP protein seen on the western blot of Tsc1 and Pten-deficient tumor tissues could not lead to the rescue of the small size of ACC RNAi tumors to the control level in vivo.

Active TORC1 is usually associated with lysosome membranes. As autophagy was seriously affected in ACC-deficient RS tumors, we also examined the integrity of the lysosomal compartment by immunolabeling Cathepsin L in control and ACC-deficient tumors. However, neither numbers nor the area of lysosomal structures were affected in ACC-silenced tumor tissues (Supplementary Fig. S3J–N).

### AMPK does not play a major role in TORC1 inactivation in ACC-deficient tumors

**Fig. 5:**
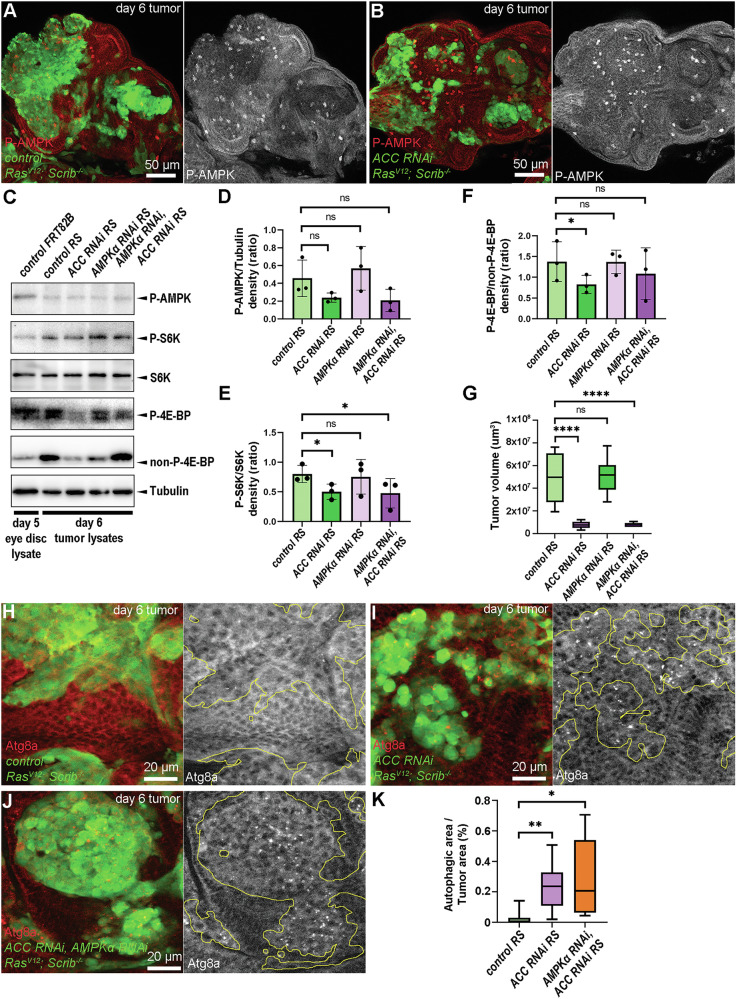
Activation of AMPK is not responsible for diminished progression of ACC-deficient RS tumors. **A**, **B** Anti-P-AMPK immunolabeling in control RS (**A**) and ACC-deficient (**B**) tumor cells (GFP + ) and in their microenvironment (GFP-). Note that the focus here was on the cytoplasmic P-AMPK signal, and the bright dots with P-AMPK antibody representing mitotic nuclei were excluded (see Supplementary Methods). **C** Western blotting of day 6 tumor samples indicates ACC and AMPKα RNAi-mediated changes in the levels of P-S6K/S6K and P-4E-BP/non-P-4E-BP ratios and P-AMPK levels. **D**–**F** Densitometry analysis of P-AMPK/Tubulin (**D**), P-S6K/S6K (**E**), and P-4EBP/non-P-4EBP (**F**) ratios based on the experiment in (**C**). Three parallel western blot experiments were analyzed (*n* = 3/genotype). Repeated Measures ANOVA, ns: non-significant *P < 0.05. **G** Analysis of the size of day 8 tumors to assay the influence of the presence and the loss of AMPK on control RS and ACC-deficient tumors. In total, 6–10 tumors/genotypes were analyzed, *n* = 7 (control RS), 8 (ACC RNAi RS), 10 (AMPKα RNAi RS), 6 (AMPKα RNAi, ACC RNAi RS). One-way ANOVA, ns: non-significant, ****P < 0,0001. **H**–**J** Immunolabeling autophagic structures – missing from control (**H**) but accumulating in ACC-deficient (**I**) RS tumor cells (GFP + )—and still present in AMPKα, ACC double-deficient (**J**) tumor tissues (GFP + ). **K** Quantification of data presented in (**H**–**J**). In total, 5–9 tumors/genotypes were analyzed, *n* = 7 (**E**), 9 (**F**), 5 (**G**). Kruskal–Wallis test, *P < 0,05; **P < 0,01. GFP+ tumor areas are encircled with yellow in grayscale panels of (**A**, **B**, **E**–**G**).

In addition to Insulin/PI3K and nutrient availability [11, 12, 25], AMPK-mediated inhibitory phosphorylation is another critical regulator of TORC1. To test whether AMPK is activated upon the loss of ACC, we immunolabeled the active, phosphorylated form of AMPK in RS tumors. Cancer cells of control RS tumors showed lower P-AMPK signal compared to the non-transformed microenvironmental cells (Fig. 5A). In contrast, ACC-deficient cancer cells did not show such a difference in P-AMPK levels (Fig. 5B). To test whether this is due to increased AMPK activity, we generated RS tumors double-deficient for ACC and the α-subunit of AMPK, by using an AMPKα RNAi transgene, we verified by qPCR and its inhibitory effect on starvation-induced autophagy in fat cells of L3 larvae (Supplementary Figs. S3A and S4A). Western blotting day 6 tumors revealed that the relative levels of P-AMPK compared to Tubulin loading control did not increase in ACC-deficient conditions (Fig. 5C, D) and loss of AMPK had only a negligible stimulatory effect on P-S6K/S6K ratios (Fig. 5C, E). Interestingly, loss of AMPK could increase the P-4EP-BP/non-P-4E-BP ratios (Fig. 5C, F) but again, potentially due to transcriptional upregulation of the total level of 4E-BP observed in this genotype (see “Discussion” for potential explanation). In line with these, tumor-specific silencing of AMPK did not restore the size of ACC-deficient tumors in comparison to control RS (Fig. 5G and Supplementary Fig. S4B, C) at day 8.

As AMPK also induces catabolism by stimulating autophagy through inactivation of TORC1 and direct phosphorylation of Atg1/ULK1 complex [13], we questioned whether AMPK may contribute to the elevated autophagy in ACC RNAi tumors (Fig. 4E, F). However, immunolabeling of autophagic structures revealed that concomitant AMPK inhibition does not reduce the number of Atg8a-positive autophagosomes in ACC-deficient cells (Fig. 5H–K). These findings suggest that the AMPK activity cannot account for the observed low TORC1 activity and elevated levels of autophagy in ACC-deficient RS tumors.

### FAs can activate TORC1 in ACC-deficient RS tumors

**Fig. 6:**
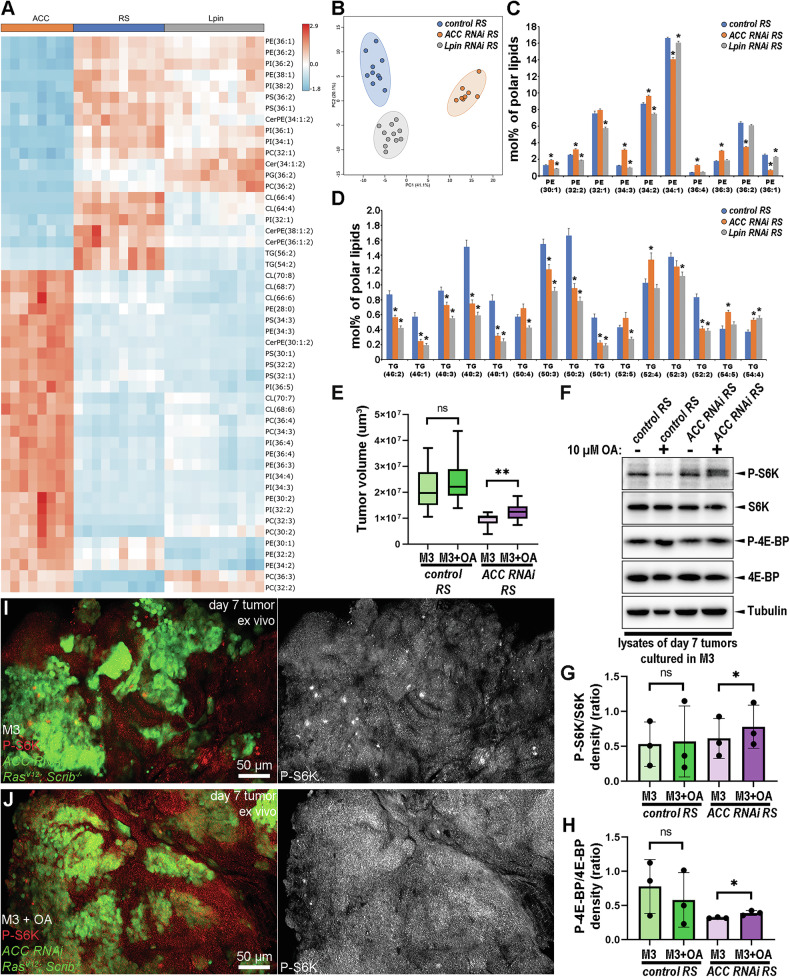
Saturated and monounsaturated FAs are critical for the progression of ACC-deficient tumors. **A**–**D** Shotgun lipidomics analysis of day 8 control, and ACC or Lpin-deficient RS tumors. Relative amounts were measured and normalized for polar lipids. **A** Cluster analysis representing the top 50 lipid species whose amount produces the highest relative changes in the analysis. **B** Principal Component Analysis reveals that lipid profiles of ACC RNAi samples are more divergent from controls than Lpin-deficient ones. **C**, **D** Diagrams representing the relative amount of the most important PE (those that are above 1%) and TG species (those that are above 0,2%). Analysis of PE profile indicates that species composed of mostly long-chained saturated or monounsaturated FAs (like PE 34:1; PE 36:1 and PE 36:2) are decreased, while those ones with 3 or 4 double bonds (that contain at least one PUFAs are significantly upregulated in ACC-deficient tumors (**C**). Analysis of TG profile shows that most of the TG species are strongly decreased in both ACC and Lpin RNAi tumors. However, those TG-s that contain at least one PUFAs (4 or more double bonds) are also significantly upregulated in ACC-deficient samples (**D**). Statistical analysis on C and D was done by comparing values from ACC RNAi RS and Lpin RNAi RS to control RS data by using Welch’s *T* test, and asterisk (*) above the columns represent significant difference (*P* < 0.05). In total, 8–11 samples/genotypes were analyzed, *n* = 10 (control RS), 8 (ACC RNAi RS),11 (Lpin RNAi RS). **E** Analysis of the size of day 7 tumors that were previously cultured in ex vivo conditions in normal M3 or oleic acid (OS) supplemented M3 media for 24 h. 10-19 samples/genotypes were analyzed, *n* = 10 (control RS M3), 10 (control RS M3 + OA), 18 (ACC RNAi RS M3), 19 (ACC RNAi RS M3 + OA). Welch’s *T* test (control RS M3 vs control RS M3 + OA) and Mann–Whitney test (ACC RNAi RS M vs ACC RNAi RS M3 + OA), ns: non-significant, **P < 0,01. **F** Western blotting to measure P-S6K/S6K and P-4E-BP/non-P-4E-BP levels in the lysates of day 7, ex vivo cultured tumors treated with or without OA. **G**, **H** Densitometry analysis of P-S6K/S6K (**G**) and P-4EBP/non-P-4EBP (**H**) ratios based on the experiment in (**F**). Three parallel western blot experiments were analyzed (*n* = 3/genotype). Paired *T* tests, ns: non-significant *P < 0.05. **I**, **J** Anti-P-S6K immunolabelings for assaying TORC1 activity in day 7, ex vivo cultured ACC RNAi RS tumor tissues (GFP + ) and their microenvironments (GFP-), treated without (**I**) or with OA (**J**).

To assay whether loss of FA synthesis causes a specific change in cellular lipid composition that may affect TORC1 activity in cancer cells, we carried out shotgun lipidomics analysis on late-staged (day 8) control and ACC-deficient RS tumors and also included Lpin-deficient ones for further analysis (Supplementary Table S1). This way, we could distinguish between the common and specific alterations in the lipid profile that were caused by cancer cell-specific perturbation of FA (*ACC RNAi*) or DG (*Lpin RNAi*) synthesis. Interestingly, comparing the lipid profiles of the three genotypes (Fig. 6A, B) revealed that ACC and Lpin-deficient samples diverged sharply from controls and from each other. Loss of ACC caused a general alteration in the FA profiles with a significant decrease in lipid species composed of saturated or monounsaturated FAs, while lipids contained at least one polyunsaturated FAs (PUFAs, with two or more double bonds) were accumulated (Fig. 6A, C, D and Supplementary Fig. S5). Considering that the enzymatic apparatus of PUFAs synthesis is lacking from *Drosophila* genome, PUFAs can only be derived from food, while saturated and monounsaturated lipids can be synthesized de novo. Hence, this FA composition reveals a massive decrease in de novo-synthesized FAs in ACC-deficient RS tumors.

Lpin-deficient samples only showed alterations in the levels of some specific lipid species, like significantly increasing PG and decreasing PE levels (Fig. 6A, C and Supplementary Fig. S5A, B, F). Finally, both Lpin- and ACC-deficient tumors showed a decrease in the amount of TGs (Fig. 6D and Supplementary Fig. S5A, H), indicating decreased TG synthesis. In addition, the relative amount of phosphatidylserine (PS), ceramide-phosphoethanolamine (CerPE), and phosphatidylcholine (PC) also altered in both enzyme-deficient genotypes (Fig. 6A and Supplementary Fig. S5A, C).

To examine whether the deficit in saturated and monounsaturated FAs can be key for TORC1 inactivation in ACC-silenced tumors, we designed an ex vivo lipid supplementation assay, where we maintained tumors (dissected from larvae on day 6) ex vivo in Insect M3 media supplemented with or without 10 µM oleic acid (OA) for 24 hours. We observed that ACC-deficient RS tumors incubated in OA supplemented media grew significantly larger (Fig. 6E)—although did not reach the size of control tumors—than those that were cultured in pure M3, while OA supplementation did not affect the growth potential of control RS tumors (Fig. 6E and Supplementary Fig. S6A–D) nor did it affect the tumor tissue/microenvironment ratios in any genotypes (Supplementary Fig. S6E). In addition, western blotting also revealed that treating ex vivo tumors with OA specifically increases the P-S6K/S6K and P-4E-BP/non-P-4E-BP ratios (Fig. 6F–H) of ACC-silenced RS ones. Finally, immunofluorescence also showed an OA-dependent increase in P-S6K signal in ACC-deficient RS ex vivo tumors (Fig. 6I, J), while P-S6K fluorescence in control RS tumors was independent of OA (Supplementary Fig. S6F, G). Overall, these findings indicate that de novo-synthesized FAs can have an important regulatory effect on TORC1 activity.

## Discussion

We demonstrated that cancer cell-specific loss of ACC, FASN1, or Lpin diminished RS tumor growth and showed the requirement of ACC- dependent FA synthesis for cancer cell survival and TORC1 activity. Although we screened most enzymes involved in neutral lipid synthesis, not all were found to be essential for tumor growth. Genetic redundancy (FlyBase lists three *Drosophila* GPAT and four DGAT paralogs) possibly explains some of this. However, Lpin represents the only PA-phosphatase ortholog. As the effect of Lpin RNAi on cancer cell apoptosis and TORC1 signaling was not addressed here, we cannot exclude that the small tumor phenotype caused by the lack of Lpin may be mediated by different mechanisms to ACC. Especially given that the loss of these two enzymes causes starkly different alterations in lipidomic profile.

Lipidomics revealed a massive decrease of de novo FA and an increase in PUFA content of membrane lipids and TGs upon ACC silencing, compared to control tumors. However, a limitation in our lipidomics data is that it cannot interpret whether this relative increase of PUFAs in ACC-deficient tumors is specific for this genotype or represents a return to the lipid composition of the normal eye disc tissue. Ex vivo experiment shows supplementation with OA—a monosaturated FA—is capable of partially overcoming FA deficit and moderately, but significantly improving the growth of ACC-deficient tumors, but not the control ones. These findings shed light on the high demand for tumors on FAs, which cannot be fulfilled by either food intake or lipid release from host tissues. Importantly, mouse xenograft experiments showed that ACC-deficient NSCLC [19] or breast cancer [18] xenografts could not progress well in the host, mostly because of upregulation of apoptosis, very reminiscent of our findings. This suggests that FA synthesis and ACC can have a conserved metabolic or signaling function required for cancer cell progression. Our lipidomic analysis of ACC-deficient tumors revealed that PUFAs are overrepresented among food- or host-derived FAs compared to those synthesized locally. Elevated PUFA levels could be disadvantageous for tumor cells, potentially by rendering them susceptible to oxidative stress associated cell death.

**Fig. 7:**
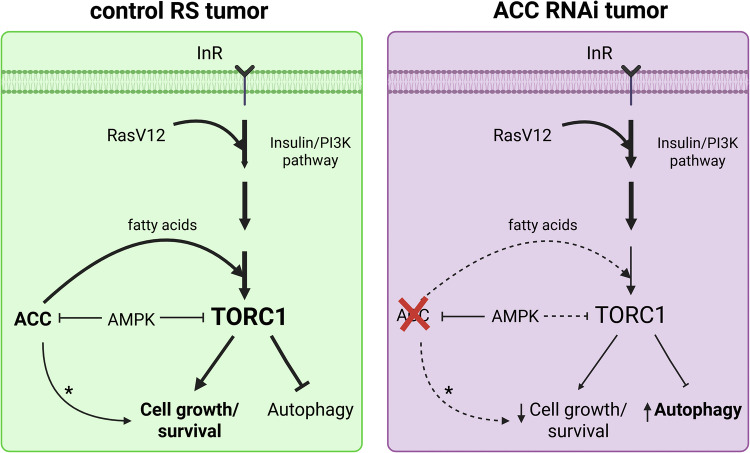
FA synthesis promotes TORC1 activity and Insulin/PI3K pathway sensitivity in cancer cells. In our proposed model, RS tumors can rapidly progress due to at least two effects of Ras^V12^. First Ras^V12^ directly induces proliferation through the MAPK pathway. Second Ras^V12^ can also stimulate TORC1-mediated cell growth and evasion of apoptosis through ectopic activation of Insulin/PI3K pathway. Our findings pointed out that high levels of TORC1 activity could be supported by ACC-mediated de novo FA synthesis. In contrast, the lack of ACC results in a critical reduction in FA synthesis, which eventually leads to TORC1 inactivation and stimulates autophagy. It is unlikely that TORC1 inactivation in ACC-depleted cells is simply due to the loss of upstream stimulatory signals, since Ras^V12^ and Insulin/PI3K pathway is still present in these cells and ectopic activation of Insulin/PI3K cannot fully restore TORC1’s activity in them. In addition, in our system, surprisingly AMPK do not, or just minimally account for reduced TORC1 signaling in ACC-deficient tumors. Instead, this situation can be rather interpreted as such that TORC1 cannot be fully stimulated by signals from the Insulin/PI3K pathway through a yet unknown mechanism upon the lack of FAs. The model allows for the possibility that ACC may affect other growth signaling pathways (marked by an asterisk) in addition to TORC1 while contributing to tumor cell progression.

Diminished TORC1 activity offers a likely explanation for the lower size and increased apoptotic and autophagic activity of ACC-deficient tumors. Our *Drosophila* model also enabled rescue experiments in vivo, which demonstrated that either reactivation of Insulin/PI3K signaling, or silencing of AMPK had minimal effects in restoring TORC1 activity and growth upon loss of ACC. However, we also observed that P-4E-BP/non-P-4E-BP and P-S6K/S6K ratios did not respond equally to activation of insulin signaling or AMPK inhibition, and differential response of these substrates for altered TORC1 activity has been reported before [26, 27]. The total 4E-BP protein level seemed highly dynamic: it decreased upon loss of ACC and increased when Insulin signaling was induced. Interestingly, dual inhibition of ACC and AMPK also gave elevated 4E-BP levels, suggesting that AMPK may contribute to the downregulation of 4E-BP expression in ACC-deficient tumors. In contrast, S6K protein levels were stable in all conditions and served as a much better proxy for measuring TORC1 activity via western blot in RS tumors. These findings indicate that TORC1 becomes less inducible by upstream Insulin/PI3K signaling upon the loss of ACC. Comparison with wild-type eye discs showed that TORC1 is hyperactivated in RS tumors likely due Ras^V12^ mediated activation of PI3K [21] and FAs may contribute to TORC1’s hyperactivity (Fig. 7). Importantly, our findings are in line with a previous *Drosophila* paper, which showed that mutation of FASN1 can decrease the overgrowth of Pten-deficient but not normal adipose tissue cells [28]. However, we also cannot exclude that, in addition to TORC1, other signaling processes driving growth may also be affected in ACC RNAi tumors (Fig. 7).

Our ex vivo experiments suggested that FAs can promote TORC1 activity, however the precise mechanism is still puzzling. Others have demonstrated that TORC1 is also regulated by its subcellular localization [11], and palmitate treatment was shown to promote its lysosomal localization and activation in cultured podocytes [29]. Although it is a tempting possibility that loss of ACC may disrupt TORC1 localization to lysosomal membranes, our observation that the lysosomal compartment remains intact weakens this hypothesis. However, considering the extensive changes in the lipid composition of ACC-deficient tumors, we cannot rule out that alteration of the membrane composition of lysosomes or other organelles may affect TORC1 activity and localization. For example, ACC1 inhibition-associated alterations in ER membrane lipid composition can induce unfolded protein response in multiple myeloma cells [30]. Importantly, a recent preprint study [31] demonstrated a link between ER stress and TORC1 inactivation, which could result in similar phenotypes to those documented here.

A recent study [32] revealed an important link between TORC1 and FA synthetic apparatus, by showing that TORC1 physically interacts with ACC and FASN both in yeast and cultured human cells, and elevated malonyl-CoA (the product of ACC) upon hyperactivation of ACC or blocking FASN can inhibit TORC1 signaling, in a FA availability independent manner. In addition, another study [33] showed that inhibition of FASN leads to mTOR malonylation and reduced TORC1 activity in endothelial cells that can be restored by downregulating ACC. Although our research resulted in a different outcome, the context is very different. We found that loss of both ACC and FASN1 caused reduced tumor size, we only investigated TORC1 activity in ACC-deficient conditions, which was dependent on FAs. However, we cannot rule out that elevated malonyl-CoA levels may also contribute to smaller sizes of FASN1 RNAi tumors.

Although cancer cells of RS tumors are highly sensitive to the integrity of the autophagic process [34, 35], they actually show only moderate autophagic activity [35]. In contrast, we observed that hyperactivation of autophagy is characteristic of ACC-deficient RS tumors, probably because autophagy may increase the cellular FA pool by degrading membrane-bound organelles. Our findings may contradict an earlier study that demonstrated that constitutively active ACC promotes autophagy in aging yeast when AMPK/Snf1 is deleted [36]. However, our observation that hyperactivated autophagy remains unaltered in ACC, AMPK double-deficient tumors suggests that induction of autophagy in these may be related to TORC1 downregulation rather than AMPK activity. Thereby, it seems likely that ACC has a complex role in autophagy, and its facilitatory or inhibitory role on autophagy is cell type and context dependent.

Overall, we demonstrated that de novo FA synthesis promotes TORC1 activity by keeping it fully inducible by upstream Insulin signaling, which points out FA synthesis as potentially promising targets in tumors with hyperactivated Insulin/PI3K pathway.

## Materials and methods

### Sample collection and biological replicates

Sample collection was primarily performed using biological replicates. In our system, each tumor is unique and can be dissected, stained, and imaged only once; thus, there is no identical second tumor from the same animal. Technical replicates were only possible for qPCR experiments (for details, see Supplementary Methods). For western blot densitometric analysis, we used three parallel biological samples per condition, each derived from 5–15 animals.

Larvae with tumors were collected randomly, otherwise no formal randomization was performed during tumor sample collection. During data analysis, images of poor quality (e.g., severely overexposed, unevenly stained in some regions, or excessively noisy, preventing accurate macro-based measurement) were considered as outliers and excluded from the analysis. Quantitative image analysis was performed under blinded conditions; investigators were unaware of the sample genotypes during data processing and measurements.

Each piece of our samples represents either a single or a cohort of individual animals. Sample sizes (n values) were chosen based on the literature and variability observed in preliminary experiments. In all cases, a minimum of five biological replicates (5 animals) were used, with a maximum of 20, limited by animal availability and experimental constraints. For all experiments, each genotype was measured at least three times, always compared to its respective control collected at the same time.

### General statistics and data analysis

All image analyses were performed using ImageJ, and resulting raw data tables were further processed using custom Python scripts to generate datasets suitable for statistical testing in GraphPad Prism 10, except for qPCR experiments (analyzed in RStudio) and lipidomic data statistics and tumor-microenvironmental tissue ratios (analyzed in Microsoft Excel). Box plots were used to visualize the data, showing the median (lines in the middle of the boxes) and interquartile range (25th–75th percentile), with whiskers extending to minimum and maximum values. Column plots display mean values with error bars representing the standard deviation (mean ± SD). Significance levels are indicated on the statistical charts as follows: ns: non-significant; *P < 0.05; **P < 0.01; ***P < 0.001; ****P < 0.0001. For simplicity, in lipidomic data visualizations, significant differences are marked with a single asterisk only (*P* < 0.05), and exact *P* values are not reported.

When comparing two groups, a two-tailed Welch’s *t* test was used for normally distributed data (verified by the Shapiro–Wilk normality test), and a two-tailed Mann–Whitney test was used for non-normal distributions. When comparing multiple groups to a control, an ordinary one-way ANOVA was applied for normally distributed data, and a Kruskal–Wallis test for non-normal distributions, both followed by Dunnett’s or Dunn’s post-hoc test, respectively, to correct for multiple comparisons. For densitometry analyses, Repeated Measures (RM) one-way ANOVA (followed by Dunnett’s post-hoc test) or paired *T* tests were performed. All statistical tests were performed as two-sided.

All statistical tests and additional methods are described in detail in the Supplementary Information, available at (*Cell Death* & *Disease*). All genotypes used are listed in Supplementary Table S2. All used antibodies were commercially available, except guinea pig anti-S6K [37], which was kindly provided by Aurelio Teleman (Heidelberg University, Germany) and are listed in Supplementary Table S3. Composition of the lipid standard mix used for MS can be found in Supplementary Table S4. All raw quantification data can be found in Supplementary Table S5.

## Supplementary information

Supplementary Information

Supplementary Fig. S1

Supplementary Fig. S2

Supplementary Fig. S3

Supplementary Fig. S4

Supplementary Fig. S5

Supplementary Fig. S6

Supplementary Table S1

Supplementary Table S2

Supplementary Table S3

Supplementary Table S4

Supplementary Table S5

Original Data

## Data Availability

All raw data is uploaded as Supplementary Information. Custom ImageJ macros and Python scripts used in this study are tied to our specific hardware and software environment but are available from the corresponding author upon reasonable request for reproducing results or academic collaboration.
