# Supplementary material for: Fatty acid synthesis supports tumor progression through facilitating the activity of TORC1 signaling
Source: Cell Death Dis. 2026 Apr 10;17(1):468. doi: 10.1038/s41419-026-08738-6 (PMC13181055; doi:10.1038/s41419-026-08738-6)
Supplement: Supplementary file 1 — Supplementary Information [file 41419_2026_8738_MOESM1_ESM.docx]

# Supplementary information

## Supplementary Methods

**Drosophila genetics**

Fruit flies were maintained in glass tubes on agar-corn flour-yeast medium, at constant 25°C. Malignant tumors were induced using the MARCM (Mosaic Analysis with Repressible Cell Marker) strategy. by using an *ey-Flp, UAS-Dcr; Act-FRT-CD2-FRT-Gal4, UAS-GFP; FRT82B tub-Gal80* genotype that were crossed with various tumor effector lines that carried a transgenic *UAS-Ras^V12^* oncogene, and loss of function mutant allele of *Scribble* (*Scrib*) cell polarity gene (*Scrib^1^*)^1^. Fat body clones were induced by “Flp-out” strategy, by crossing RNAi lines to a *hs-Flp; 3xmCherry-Atg8a, UAS-GFP*; *Act-FRT-CD2-FRT-Gal4,* UAS-Dcr2, where the promoter of hs-Flp was “leaky” and randomly activated in a small subset of cells^2^. For experiments on starved fat bodies, early L3 (day 4) larvae were collected from the food and were starved in PBS for 3 h before dissection, for well-fed condition larvae were kept on the fly food until dissection. All mentioned transgenic and mutant stocks were obtained from Bloomington Drosophila Stock Centre (Indiana, US) except *UAS-ACC[KK102082]* and *UAS-FASN1[GD29349]* RNAi lines that were ordered from Vienna Drosophila Research Centre. The detailed description of the genotypes used regarding each experiment are listed in **Supplementary Table S2**.

**Tumor volume analysis**

All crosses were maintained in the same tray, flipped at the same time, and larvae were dissected at a consistent time of the day and control tumors were always collected in parallel to the RNAi treated ones. Tumors were dissected from larvae in PBS and fixed in 4% formaldehyde-PBS for 30 minutes. To remove the fixative, samples were rinsed two times and washed for 15 minutes in PBS. Then tumors were mounted in glycerol. The whole volume of tumors was scanned through by optical sectioning (10x magnification Z-stack function, with 3 micron slice intervals) by using the already mentioned Zeiss AxioImager M2 fluorescence microscope.

For determination of tumor–microenvironment tissue ratios, eye discs carrying GFP-positive tumors were incubated in Hoechst 33342 solution (Thermo Scientific; final concentration 0.05 mg/mL, diluted in PBS) for 10 minutes at room temperature, at the end of the standard tumor volume measurement protocol and immediately prior to imaging.

**Lipid staining**

From all genotypes, 6-8 eye discs/tumors of 6 and 7-day-old larvae, or fat bodies of well-fed early L3 (day 4) larvae were dissected in PBS solution and fixed in 4% formaldehyde-PBS for 30 minutes on rotary shaker. To remove the fixative, samples were rinsed twice and washed for 15 minutes in PBS buffer. Then samples were incubated in PBS containing monodansylpentane/AUTODOT (Abcepta) fluorescent lipophilic dye (with 1:500 dilution) overnight at 4°C on orbital shaker. The next day, the staining solution was removed by 2x rinsing and 15 minutes washing in PBS. Finally, the samples were mounted in glycerol and imaged by Zeiss AxioImager M2 fluorescence microscope equipped by ApoTome2 (Zeiss) confocal unit and Orca-Flash 4.0 LT3 digital sCMOS camera (Hamamatsu Photonics).

**Ex vivo culturing**

The following incubation media was prepared and warmed to room temperature before the experiment: Shields and Sang’s M3 insect medium (Merck) that contained Penicillin-Streptomycin antibiotics (diluted in 1:100) and optionally supplemented by 10 µM oleic acid (#O3008, Merck). To prevent any contamination, sterile 24-well plate was used for all experiments and larvae and forceps tips were washed in 70% ethanol before dissection. For the assay, control RS and ACC RNAi RS tumors were dissected in M3 medium, and 10-15 tumors per genotype were incubated in 500-500 µl of normal or oleic acid containing M3 medium for 24 hours at 25°C, protected from direct light. Animals were randomly assigned to either M3 or oleic acid–supplemented M3 medium. After incubation, tumor sizes were assessed using the volumetric measurement or the immunolabelling protocol described above. For Western blot analyses, instead of fixation, samples were processed according to the protocol used for Western blot sample preparation.

**Immunolabellings**

On the first day of fluorescent immunolabeling, 6-8 developing eyes were dissected from 6- or 7-day-old larvae (or collected from the ex vivo culturing media, in the case of ex vivo experiments) of control RS and RS ACC genotypes and fixed in 4% formaldehyde-PBS solution for 20 minutes. After washing three times for 5 minutes each in 0.5% Triton X100-PBS solution (PBT) to remove the fixative, primary antibodies **(Supplementary Table S3)** were added in PBT containing 5% fetal bovine serum (FBS PBT). The samples were then incubated overnight at 4°C on orbital shaker. On the second day, after washing three times for 5 minutes in PBT, samples were incubated with secondary antibodies dissolved in FBS PBT for 3 hours at room temperature, in dark chamber. After removing the secondary antibodies, samples were washed two times in PBT for 5 minutes and then twice in PBS for 5 minutes. The samples were then mounted in glycerol and a Z-stack was acquired for each sample using a fluorescence microscope.

Regarding P-AMPK immunolabelings it is important to note that in addition to its cytoplasmic staining some nuclei were also stained strongly with anti-P-AMPK. As the antibody we used was shown to detect P-AMPK on the mitotic spindle in multiple species^3,4^, these bright dots likely represent mitotic nuclei. In this study we focused on cytoplasmic staining only.

**Western blotting**

Tumors from 6-day-old larvae, or ex vivo tumors collected on day 7 after 24 h incubation in M3 media, were collected in Eppendorf tubes containing lysis buffer (50 mM Tris-HCl, 150 mM NaCl, 1% Triton X-100, 5 mM EDTA) supplemented with protease (PMSF 1:100, leupeptin 1:1000, aprotinin 1:1000) and phosphatase (NaF 1:100, Na₃VO₄ 1:100) inhibitors and kept on ice. Samples were homogenized using plastic pestles, and protein concentrations were measured by standard Bradford assay. After addition of Laemmli loading buffer, samples were boiled for 5 minutes and stored at −20 °C until further use.

For western blot analysis, equal amounts of protein (7.5 μg/sample) were loaded onto 13% polyacrylamide gels and after the electrophoresis (Mini-Protean Tetra Cell device, Bio-Rad) the separated proteins were transferred to a PVDF membrane by using a Trans-Blot Turbo semidry blotting device (Bio-Rad). Membranes were washed three times in TBS containing 0.1% Tween-20 (TBST) for 5 minutes each and then blocked for 1 hour in 0.5% casein. Membranes were incubated overnight at 4 °C in a 1:1 TBST-casein solution containing primary antibodies (see Supplementary Table S3). Next day, the primary antibody was removed by 3x washing for 10 minutes in TBST. Then, the membranes were incubated for one hour at room temperature in 1:1 TBST-Casein that contained horseradish peroxidase (HRP)-conjugated secondary antibodies. Secondary antibodies were removed by 3×10 minute wash with TBST and membrane was developed by chemiluminescent HRP substrate solution and imaged by using a Chemidoc Imaging System (Bio-Rad) device.

All used antibodies were commercially available, except guinea pig anti-S6K^5^, which was kindly provided by Aurelio Teleman (Heidelberg University, Germany). The details of antibodies used are described in **Supplementary Table S3**. Uncropped versions of western blot membranes can be found as **Original Data** file**.**

**Shotgun lipidomics**

To minimize contamination, day 8 larvae were first rinsed in ethanol, and then washed in PBS. Three tumors/sample were dissected in sterile PBS and were carefully collected using dissection needles to minimize excess fluid and immediately transferred into empty Eppendorf tubes, which were then flash-frozen in liquid nitrogen. Then samples were kept on -80^o^C until further analysis. The entire lipidomics dataset is available in **Supplementary Table S1**.

Lipidomic standards were from Avanti Polar Lipids (Alabaster, AL, USA) **(Supplementary Table S4)**. Solvents for extraction and mass spectrometry (MS) analyses were Optima LC-MS grade (Thermo Fisher Scientific, Waltham, MA, USA) and liquid chromatographic grade (Merck, Darmstadt, Germany). All other chemicals were the best available grade purchased from Sigma (Steinheim, Germany) or Merck (Darmstadt, Germany).

Lipid species were annotated with sum formulas according to the shorthand notation for lipid structures^6^. For glycero(phospho)lipids, e.g., PE (34:1), the total numbers of carbons followed by double bonds for all chains are indicated. For sphingolipids, the sum formula like CerPE(34:1:2) specifies first the total number of carbons in the long chain base and FA moiety, then the sum of double bonds in the long chain base and the FA moiety, followed by the sum of hydroxyl groups in the long chain base and the FA moiety.

MS analyses were performed on an Orbitrap Fusion Lumos instrument (Thermo Fisher Scientific, Bremen, Germany) equipped with a robotic nanoflow ion source (TriVersa NanoMate, Advion BioSciences, Ithaca, NY, USA) using chips with a spraying nozzle diameter of 5.5 µm. The back pressure was set at 1 psi. The ionization voltages were +1.3 kV and −1.9 kV in positive and negative modes, respectively, whereas it was +1.5 kV in acquisitions with polarity switching. The temperature of the ion transfer capillary was 260 °C. Acquisitions were performed at mass resolution Rm/z 200 = 240 000 in full scan mode. In the polarity switching method, spectra were acquired within the mass range of m/z 400–1300 from 0.2 to 0.6 min in the negative and from 0.8 to 1.2 min in the positive polarity mode. Phosphatidylethanolamine (PE), phosphatidylinositol (PI), phosphatidylserine (PS), phosphatidic acid (PA), phosphatidylglycerol (PG), cardiolipin (CL), and the lyso derivatives LPC, LPE, and LPI as well as ceramide (Cer), hexosyl ceramide (HexCer), and ceramide phosphoethanolamine (CerPE) were detected and quantified using the negative ion mode, whereas phosphatidylcholine (PC), diacylglycerol (DG), triacylglycerol (TG), and ergosteryl ester (EE) were detected and quantified using the positive ion mode.

For lipidomic measurements, 3 tumors per sample type were collected, immediately fresh-frozen in liquid nitrogen, and stored at −80 °C. For lipid extraction the samples were subjected to a one-phase methanolic lipid extraction. The tumors were sonicated, depending on the relative tumor size, in 150 – 300 μL methanol containing 0.001% butylated hydroxytoluene (as an antioxidant) in a bath sonicator for 5 min, then shaken for 5 min and centrifuged at 10 000 g for 5 min. The supernatant was transferred into a new Eppendorf tube and stored at −20 °C until MS analysis.

For MS measurements, 8-12 μL lipid extract was further diluted with 110 μL infusion solvent mixture (chloroform:methanol:iso-propanol 1:2:1, by vol.), which was spiked with an internal standard mix (Avanti Polar Lipids, Alabaster, AL, USA; **Supplementary Table S4**). Next, the mixture was halved, and 5% dimethylformamide (additive for the negative ion mode) or 3 mM ammonium chloride (additive for the positive ion mode) were added to the split sample halves. 10 μL solution was infused and data were acquired for 2 min.

Raw MS spectra were converted to platform-independent mzML files, and lipid species were identified by LipidXplorer software (Herzog et al., 2011). Identification was made by matching the m/z values of their monoisotopic peaks to the corresponding elemental composition constraints. The mass tolerance was set to 3 ppm. Data files generated by LipidXplorer queries were further processed by self-developed Microsoft Excel macros. Quantification was made by comparing integrated MS1 signal intensities with those of the internal standards.

**Quantitative PCR analysis for RNAi knockdown validation**

Tumors were dissected in PBS, transferred into TRI Reagent® solution (Zymo Research; R2050-1–50), and homogenized. Total RNA was extracted using the Direct-zol™ RNA MiniPrep Kit (Zymo Research, R2050), which includes DNase treatment. cDNA synthesis was carried out with the RevertAid First Strand cDNA Synthesis Kit (Thermo Fisher Scientific; K1621). Quantitative PCR was performed on a LightCycler 96 instrument (Roche Molecular Systems) using the PowerUP^TM^ SYBR^TM^ Green Master Mix (appliedbiosystems; A25742). Cycling conditions were as follows: initial denaturation at 95 °C for 600 s; 45 cycles of 95 °C for 10 s, 58 °C for 10 s, and 72 °C for 20 s; melting at 95 °C for 10 s, 65 °C for 60 s, and 97 °C for 2 s; and final cooling at 40 °C for 30 s. Three biological replicates and two technical replicates were analyzed. Data points were excluded if the Cq (Quantification Cycle) values differed by more than 0.5. The mathematical model^7,8^ that was used to analyze raw qPCR data can be found in **Supplementary Table S5.**

Quantitative PCR was performed using the following primer pairs:

- **rpl32 (internal control)**:
- Forward: 5′-GCT AAG CTG TCG CAC AAA TGG-3′
  Reverse: 5′-GTA GCC AAT GCC TAG CTT GTT C-3′
- **Ampk**
  Forward: 5'-AAC CTA AAG CTC TTC CGC CA-3'
  Reverse: 5'-GCA ATA GTC CAC GCC AGA GA-3'
- **Gpat4**
  Forward: 5'-GGG GCT TCC TCT CAT TTC TCA-3'
  Reverse: 5'-CGT CGT CCA CTA GTT GCA CT-3'
- **Mdy**Forward: 5'-TTT GGC TGT GGA GAA GGG TC-3'
  Reverse: 5'-ACG GCA CCA CAT ATT CGT CT-3'
- **Mino**Forward: 5'-CCT GAA ATC AAA CTA CGG CCT C-3'
  Reverse: 5'-CGT ACA AGG AGG AGG TGG AC-3'
- **Pten**Forward: 5'-AGC CAC AGA AAA TGC AAA GCC-3'
  Reverse: 5'-TTT GTT AAC TGT TCC ATC GGA CTC-3'
- **Tsc1**
  Forward: 5'-GAC GGA GCA AAA GCG ACT TC-3'
  Reverse: 5'-TGT CAG TTC TGT CCG TGT CC-3'

**Quantification and Statistical Methods**

*Determination of tumor volume*

To quantify tumor volumes Z-stack images were processed in ImageJ. A custom macro was applied that was built up the following steps: Images were denoised by Gaussian blur 3D function, then a mask was created for each image slice to identify tumor areas based on appropriate color intensity in the green channel by using the built-in “Otsu” auto-thresholding model. Then, 3D objects were reconstructed from these masked images, representing tumor regions, and their volumes were measured by 3D objects counter function. By summing these volumes, the total tumor volume was obtained.

*Determination of tumor–microenvironment tissue ratios:*

To determine the ratio of tumor to microenvironmental tissue, we developed an ImageJ macro script that measured the GFP (labeling tumor tissue) and the Hoechst (labeling the entire eye disc) channels separately. For GFP, the previously established Otsu threshold was applied, whereas for Hoechst, the optimal built-in threshold was manually selected for each image (tipically, either the “Triangle” or “Li” auto-tresholding model provided the best segmentation). Microenvironmental tissue area was calculated by subtracting the GFP-positive tumor area from the total Hoechst-stained tissue area. These values were averaged in Excel and represented as percentages for comparison, and the tumor-to-whole tissue ratios were compared in GraphPad Prism

*Quantification of lipid and immunofluorescent stainings*

Images for each staining type were analyzed in ImageJ, with specific macros (see below). In parallel the area of GFP positive tumor tissues were also measured. Then the extracted values were aggregated and normalized to the total area of GFP+ tumor tissues by using a Python script. The processed values were then analyzed in GraphPad Prism. For statistical analysis Welch’s unpaired T-tests or Mann-Whitney tests were applied in case of datasets with normal or non-normal distribution respectively.

***For MDH lipid staining analysis***, a macro was used to compare the total area of lipid droplets in the GFP-positive tumor areas across the different genotypes. The “Otsu” auto-thresholding model was applied for measuring GFP-positive areas, while the “MaxEntropy” auto-thresholding model was used for masking the MDH channel. Then, the masked MDH channel was quantified in 2D using the “Analyze Particles” plugin.

***For quantification of apoptotic areas***, a similar approach to lipid staining analysis was applied. However, threshold was set by a custom script written in ImageJ, which calculated the masked area based on the characteristic size of the stained regions, each image mask was validated manually before quantification. The “Otsu” auto-threshold was still applied to mask the GFP-positive tumor areas. The masked areas were then quantified using the “Analyze Particles” plugin.

***For P-H3 immunostaining***, a custom macro was developed to compare the number of dividing cell nuclei in GFP-positive areas between control RS and ACC RNAi RS genotypes. “Otsu” auto-thresholding method was again applied to GFP-positive areas, while the “Moments” thresholding model was used for P-H3 staining. Two image slices (specifically, the third slice from both the top and bottom of each Z-stack image) were quantified using the “Analyze Particles” plugin, and the values from both slices were averaged for further calculations.

***For immunostaining of autophagic structures***, GFP-positive tumor areas were masked using the “Default” auto-threshold, while the Atg8a channel was masked with the “Moments” auto-threshold model. Each Z-stack image consisted of seven slices (with 0,35 μm slice width) and measurements were taken from the middle three slices (3, 4, and 5) to ensure accurate quantification. The autophagic structures within tumor areas were analyzed on these three slices using the “Measure” command, and the values were then averaged to obtain a final measurement.

***For the analysis of Cathepsin-L–labeled lysosomal structures***, the GFP-positive tumor areas were processed by applying a Gaussian blur (σ = 2) to the green channel, followed by manual thresholding. ROIs corresponding to the tumor area were generated for each image. The far-red channel was then processed by manually thresholding Cathepsin L-positive puncta. The tumor ROIs were applied to this channel, and the size and number of Cathepsin L-positive puncta were quantified using the “Analyze Particles” function. GFP-positive tumor areas were measured using the “Measure” function after ROI selection. Puncta number per tumor area, average puncta size per tumor, and the cathepsin compartment area relative to tumor area were used for comparison between the genotypes. Statistical significance was assessed using Welch’s t-test for each dataset.

*Densitometric analysis of western blot results*

Western blot images were analyzed in ImageJ using the *Gels* analysis functions. When necessary, images were inverted to enhance the contrast between bands and background. For each lane, rectangular ROIs of consistent size were drawn around the protein bands, including adjacent background regions to allow background subtraction. Band intensities were quantified using the *Analyze → Gels* function, generating density profiles for each lane and calculating peak areas corresponding to individual bands. For normalization, phosphorylated protein levels were quantified relative to their corresponding total or reference controls: P-S6K intensities were normalized to total S6K, P-4E-BP to non-phosphorylated-4E-BP, and P-AMPK to Tubulin, as no Drosophila-reactive total AMPK antibody is available. The resulting ratios were statistically compared between genotypes using GraphPad Prism.

## Supplementary References

1. Dillard, C., Reis, J.G.T. & Rusten, T.E. Tumors: A Cooperative Oncogenesis Model Fueled by Tumor/Host Interactions. *Int J Mol Sci* **22**(2021).

2. Hegedus, K. *et al.* The Ccz1-Mon1-Rab7 module and Rab5 control distinct steps of autophagy. *Molecular Biology of the Cell* **27**, 3132-3142 (2016).

3. Tripodi, F., Fraschini, R., Zocchi, M., Reghellin, V. & Coccetti, P. Snf1/AMPK is involved in the mitotic spindle alignment in Saccharomyces cerevisiae. *Sci Rep* **8**, 5853 (2018).

4. Vazquez-Martin, A., Oliveras-Ferraros, C. & Menendez, J.A. The active form of the metabolic sensor: AMP-activated protein kinase (AMPK) directly binds the mitotic apparatus and travels from centrosomes to the spindle midzone during mitosis and cytokinesis. *Cell Cycle* **8**, 2385-98 (2009).

5. Hahn, K. *et al.* PP2A regulatory subunit PP2A-B' counteracts S6K phosphorylation. *Cell Metab* **11**, 438-44 (2010).

6. Liebisch, G. *et al.* Shorthand notation for lipid structures derived from mass spectrometry. *J Lipid Res* **54**, 1523-1530 (2013).

7. Pfaffl, M.W. A new mathematical model for relative quantification in real-time RT-PCR. *Nucleic Acids Res* **29**, e45 (2001).

8. Varga, V., Szinyákovics, J., Bebes, A., Szikszai, F. & Kovács, T. Role of Hemocytes in the Aging of Drosophila Male Germline. *Cells* **14**(2025).

## Supplementary Figure and Table Legends

### Supplementary Figure Legends

**Supplementary Fig. S1. Tumor tissue/microenvironment ratio progressively grows in control RS but not ACC RNAi RS tumor containing eye discs. (A-C)** In eye discs containing early staged (day 6) **(A)** tumors. Day 7 **(B)** and day 8 **(C)** the tumor tissue begins to overgrow the microenvironment causing a shift of the tumor tissue/microenvironment ratio. In contrast ACC RNAi RS tumors do not have this growth differential and the tumor tissue/microenvironment ratios are stabilized at a lower level in later stages. Mean volumetric ratios of tumor tissues (green) and microenvironments (blue) are represented (in %). **(A’-C’)** Quantifications of relative tumor tissue/eye disc volumetric ratios. Eye discs from 7-12 larvae/genotypes were analyzed n= 9 (control RS day 6), 7 (ACC RNAi RS, day 6), n= 9 (control RS day 7), 12 (ACC RNAi RS, day 7), n= 9 (control RS day 6), 9 (ACC RNAi RS, day 8). Welch’s T-tests, ns: non-significant, ****: p<0001.

**Supplementary Fig. S2: Additional genetic screen data.** **(A-C)** Representative images at day 7 of mino **(A)**, Gpat4 **(B)** and mdy **(C)** deficient RS tumors from the RNAi screen. Quantification of respective tumor size data is shown on **A’-C’**. Note that each RNAi dataset was compared to a dedicated control RS dataset that was collected and measured in parallel. 7-9 tumors were analyzed/genotypes, n=8 (control RS) and 9 mino RNAi RS) in A, 7 (control RS) and 8 (Gpat4 RS) in B, 7 (control RS) and 7 (mdy RNAi RS) in C. Mann-Whitney test (A), Welch’s T-test (B, C), ns: non-significant. **(D)** qPCR measurement of the mRNAi levels of mino, Gpat4 and mdy genes in RS tumors expressing mino, Gpat4 and mdy RNAi transgenes respectively. For each genotype, the qPCR analysis was performed using 3 biological and 2 technical replicates, with each sample containing 5 tumors, n=6 (control RS), 6 (Gpat4), 5 (mdy), 6 (mino), one outlier was excluded in the mdy RNAi RS dataset Mann-Whitney test, **: p<0,01, ***: p<0,001 **(E-G)** Staining lipid droplets by Monodansylpentane (MDH) in genetic mosaic fat bodies of well-fed early L3 larvae. Gal4 expressing cell clones that also express Gpat4 **(E)**, mino **(F)** and mdy **(G)** RNAis are represented by GFP positivity, while the neighbouring GFP and Gal4 negative cells are serving as controls. The decrease of LD size in mdy RNAi cells reports the efficiency of the mdy transgene, while LDs were unaltered in Gpat4 and mino RNAis, suggesting that these two enzymes may act redundantly.

**Supplementary Fig. S3: The size of ACC RNAi RS tumors is insensitive for hyperactivation of Insulin/PI3K pathway. (A)** Validation of Pten, Tsc1 and AMPKα RNAi transgenes by qPCR carried out on control RS, Tsc1 RNAi RS and Pten RNAi RS tumor samples. For each genotype, the qPCR analysis was performed using 3 biological and 2 technical replicates, with each sample containing 5 tumors, n=6 (control RS), 5 (Pten), 6 (Tsc1), 6 (AMPKα), one outlier was excluded in the Pten RNAi RS dataset. Mann-Whitney test, ***: p<0,001. **(B, C)** Validation of Pten and Tsc1 RNAi transgenes by testing their effect on starvation induced autophagy, in genetic mosaic fat bodies of early L3 larvae starved for 3 h. Both Tsc1 **(B)** and Pten RNAi **(C)** cell clones (expressing Gal4 and represented by GFP positivity) show larger sized nuclei and smaller and less autophagic structures (autophagosomes and autolysosomes) that are positive for 3xmCherry-Atg8a autophagic reporter compared to the neighboring GFP negative (not expressing Gal4) control cells. These phenotypes indicate that TORC1 is activated in these RNAi cell clones. **(D-I)** Representative images about late staged (day 8) RS control **(D)**; ACC RNAi RS **(E)**; Pten RNAi RS **(F)**; Pten RNAi, ACC RNAi RS **(G)**; Tsc1 RNAi RS **(H)**; Tsc1 RNAi, ACC RNAi RS tumors **(I)**. The quantifications of data from these experiments can be seen on **Fig. 4H**. **(J, K)** Immunolabeling of lysosomal hydrolase Cathepsin L does not indicate any major alterations in the morphology of lysosomal compartment in day 6 control RS **(J)** and ACC RNAi RS **(K)** tumors. **(L-N)** Quantification data of lysosome number **(L)**, size **(M)** and relative area of lysosomal compartment **(N)** in GFP positive tumor tissues. 5 tumors were analyzed/genotypes, n=5 (control RS), 5 (ACC RNAi RS) in panels L-N. Welch’s T-test (L-N) ns: non-significant.

**Supplementary Fig. S4. Efficient knockdown of AMPK is not increasing ACC RNAi RS tumor size. (A)** Validation of AMPKα RNAi transgene by testing its effect on starvation induced autophagy, in genetic mosaic fat bodies of early L3 larvae starved for 3 h. Gal4 expressing cell clones that also express AMPKα RNAi are represented by GFP positivity, while the neighbouring GFP and Gal4 negative cells are serving as controls. AMPKα RNAi cell clones (expressing Gal4 and represented by GFP positivity) show less and smaller autophagic structures (autophagosomes and autolysosomes) that are positive for 3xmCherry-Atg8a autophagic reporter compared to the neighboring GFP negative (not expressing Gal4) control cells. **(B, C)** Representative images about late staged (day 8) AMPK RNAi RS **(B)** and AMPK RNAi, ACC RNAi RS **(C)** tumors**.**

**Supplementary Fig. S5: Detailed lipid profiles of control and ACC or Lpin deficient RS tumors. (A)** Diagram representing the relative amounts of the major lipid classes. **(B-H)** Diagrams representing the relative amounts of all species of PE **(B)**, PC **(C)**, PS **(D)**, PI **(E)**, PG **(F)**; PA **(G)**; TG **(H)**. Statistical analysis on A-H was done by comparing values from ACC RNAi RS and Lpin RNAi RS to control RS data by using unpaired Welch’s T-test and asterisks (*) above the columns represent significant difference (p<0,05). 8-11 samples/genotypes were analyzed, n=10 (control RS), 8 (ACC RNAi RS),11 (Lpin RNAi RS). PC: Phosphatidylcholine; LPC: Lyso-phosphatidylcholine; LPE: Lyso-phosphatidylethanolamine; PE: Phosphatidylethanolamine; LPI: Lyso-Phosphatidylinositol; PI: Phosphatidylinositol; PS: Phosphatidylserine; PG; PA: Phosphatidic acid; CL: Cardiolipin; CerPE: Ceramide-phosphoethanolamine; Cer: Ceramide; HexCer: Hexosylceramide; DG: Diacylglycerol; TG: Triacylglycerol; EE: Cholesteryl-ester

**Supplementary Fig. S6: Oleic acid treatment partially restores the growth of ACC deficient tumors.** Representative images about control RS **(A, B)** and ACC RNAi RS **(C, D)** tumors dissected on day 6 and incubated ex vivo in M3 media alone **(A, C)** or supplemented with 10 µM oleic acid (OA) **(B, D)**. The quantifications of data from these experiments can be seen on **Fig. 6E**. **(E)**  Quantifications of volumetric ratios of tumor tissue and microenvironment in day 7 tumors cultured in ex vivo conditions for 24 h. Similar to control RS tumors, no substantial alterations in tumor tissue/microenvironment ratios can be detected between ACC RNAi RS tumors incubated in control or oleic acid (OA) supplemented M3 media. Mean volumetric ratios of tumor tissues (green) and microenvironments (blue) are represented (in %). **(E’)** Quantifications of relative tumor tissue/eye disc volumetric ratios. Eye discs from 8-11 larvae/genotypes were analyzed, n= 8 (control RS, M3), 11 (control RS, M3 + OA), 10 (ACC RNAi RS, M3), 9 (ACC RNAi RS, M3 + OA). Welch’s T-tests, ns: non-significant. **(F, G)** Anti-P-S6K immunolabellings for assaying TORC1 activity in day 7, ex vivo cultured control RS tumor tissues (GFP+) and their microenvironments (GFP-), treated without **(F)** or with OA **(G)**.

### Supplementary Table Legends

**Supplementary Table S1: The entire dataset of shotgun lipidomics analysis**

**Supplementary Table S2: Detailed genotypes in each Figures**

**Supplementary Table S3: List and details of the used antibodies**

**Supplementary Table S4: List of internal standards used in lipidomics analysis**

**Supplementary Table S5: Raw quantitative data**
