# Supplementary figures and images for: Fatty acid synthesis supports tumor progression through facilitating the activity of TORC1 signaling
Source: Cell Death Dis. 2026 Apr 10;17(1):468. doi: 10.1038/s41419-026-08738-6 (PMC13181055; doi:10.1038/s41419-026-08738-6)

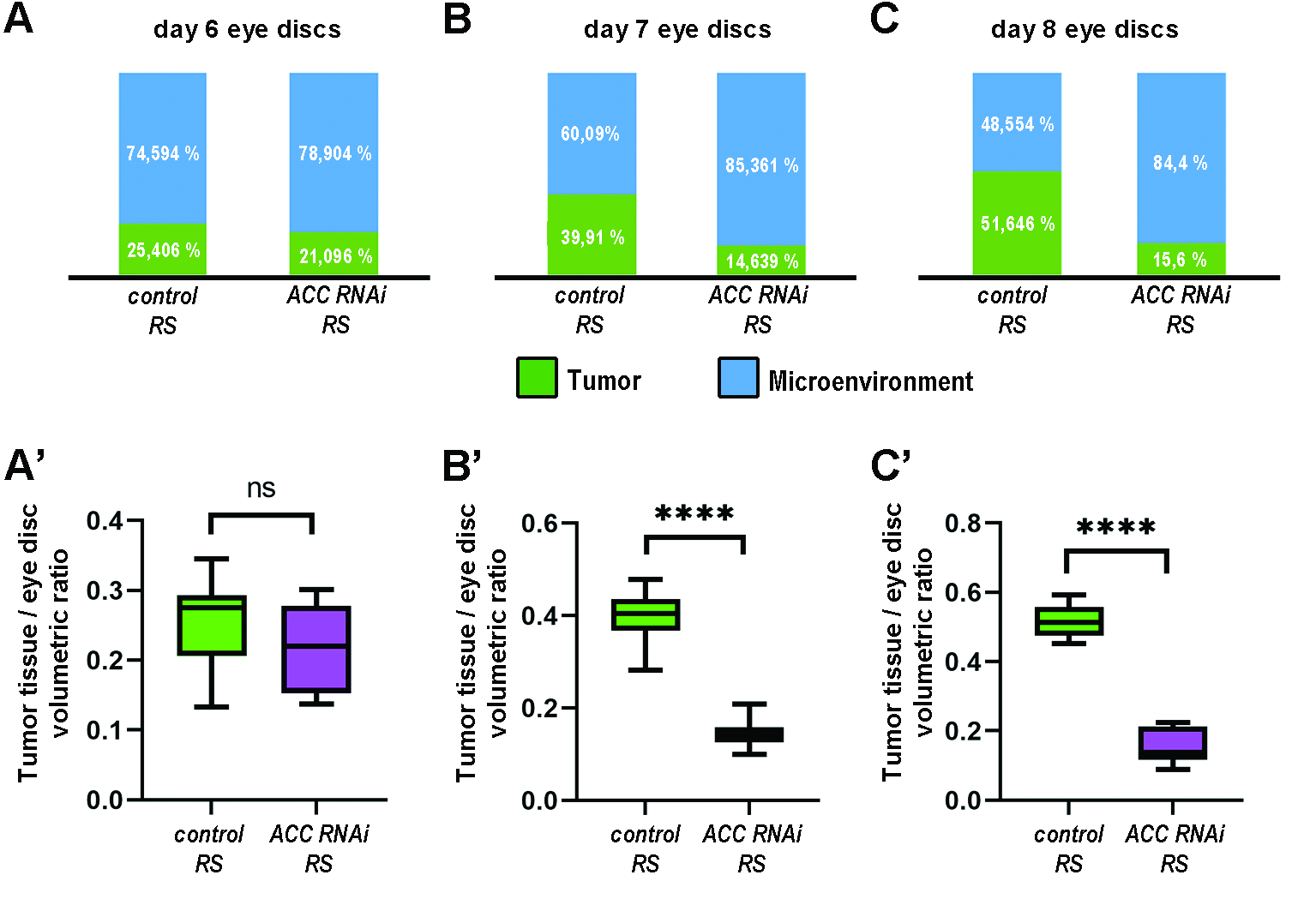

Supplement: Supplementary file 2 — Supplementary Fig. S1 [file 41419_2026_8738_MOESM2_ESM.tif]

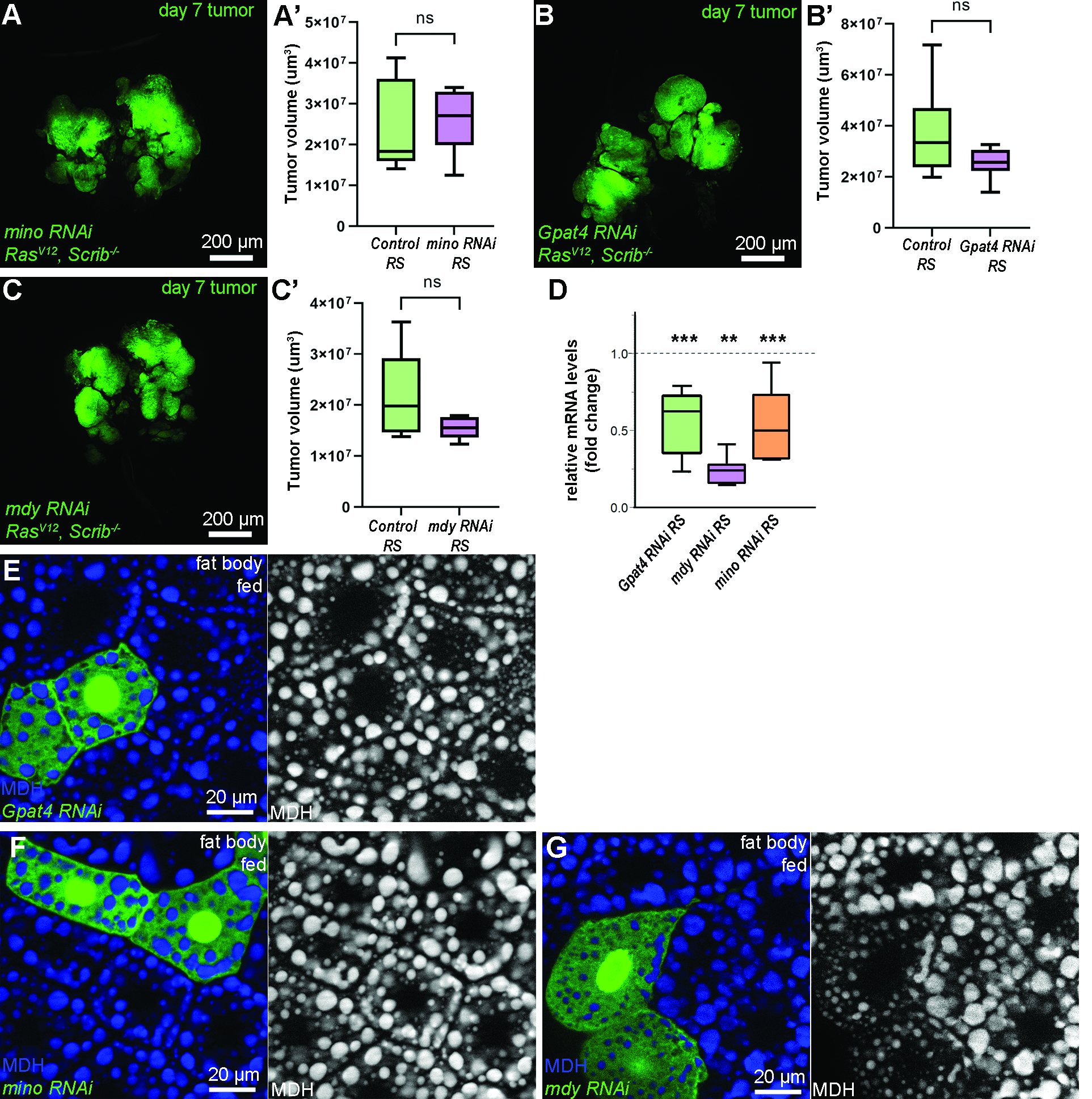

Supplement: Supplementary file 3 — Supplementary Fig. S2 [file 41419_2026_8738_MOESM3_ESM.tif]

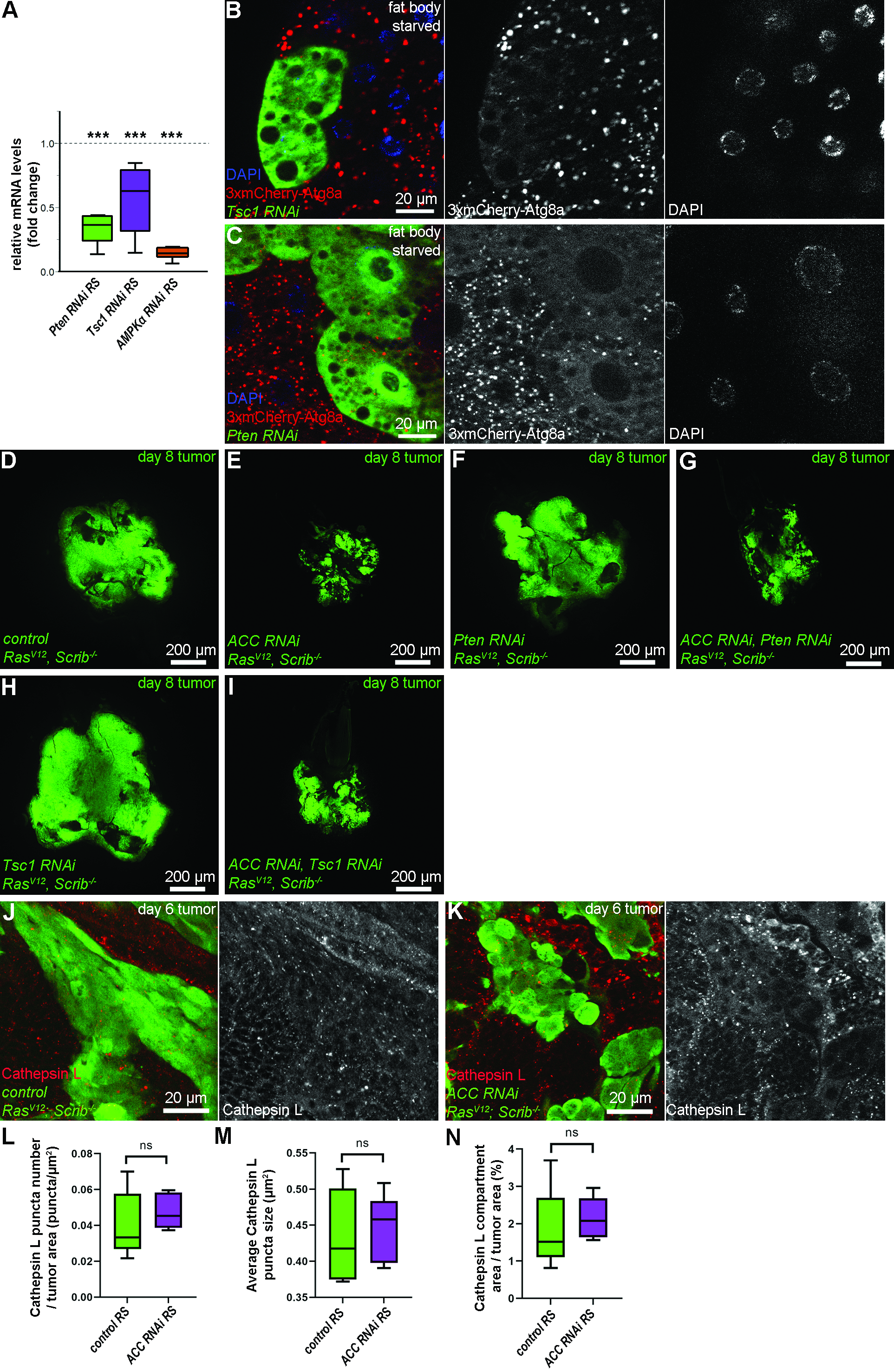

Supplement: Supplementary file 4 — Supplementary Fig. S3 [file 41419_2026_8738_MOESM4_ESM.tif]

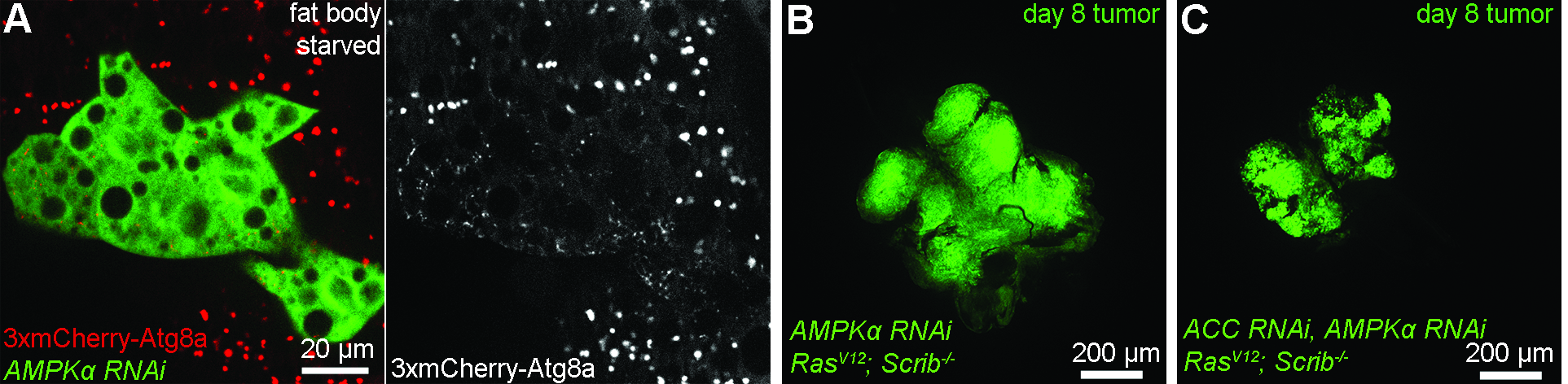

Supplement: Supplementary file 5 — Supplementary Fig. S4 [file 41419_2026_8738_MOESM5_ESM.tif]

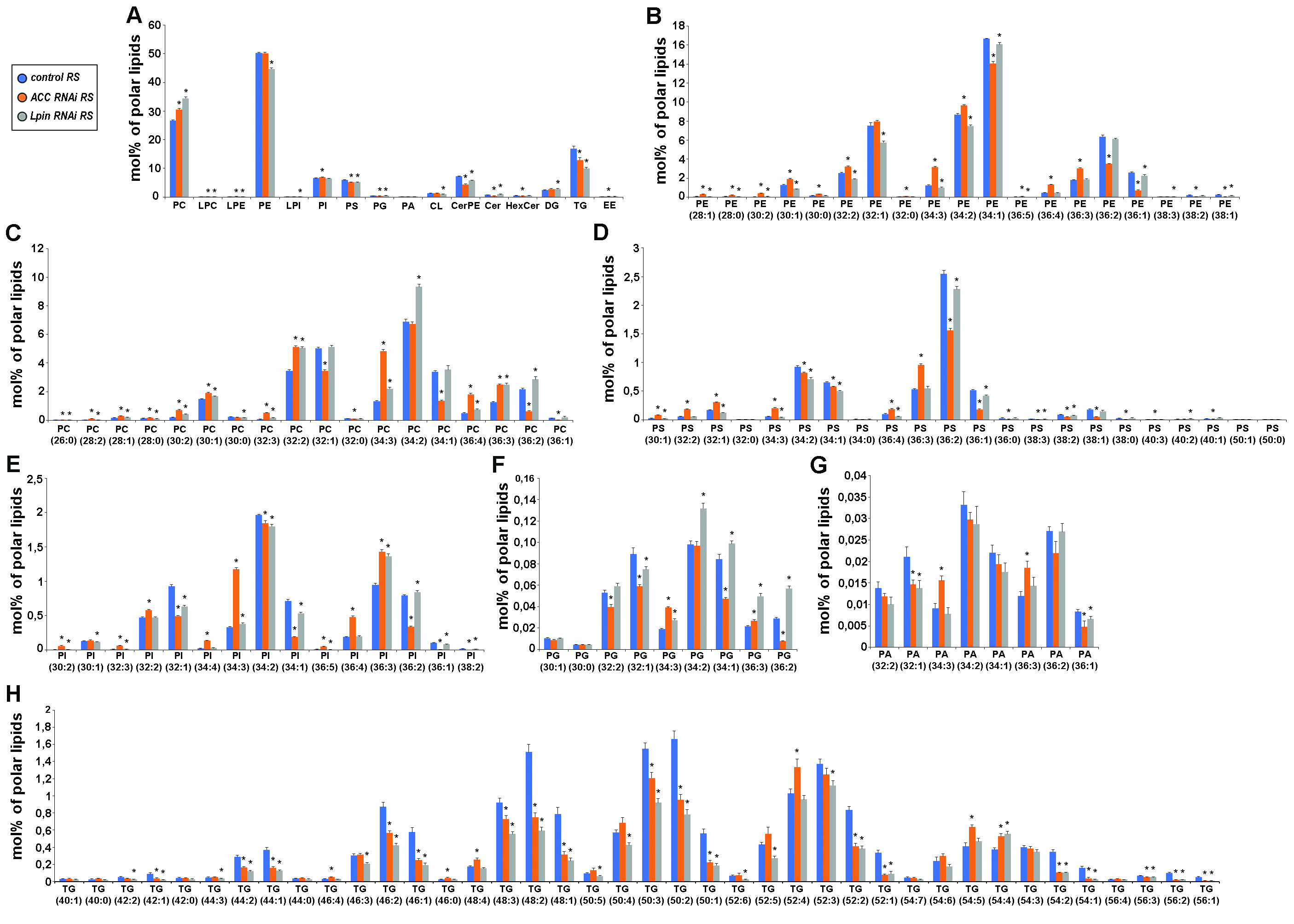

Supplement: Supplementary file 6 — Supplementary Fig. S5 [file 41419_2026_8738_MOESM6_ESM.tif]

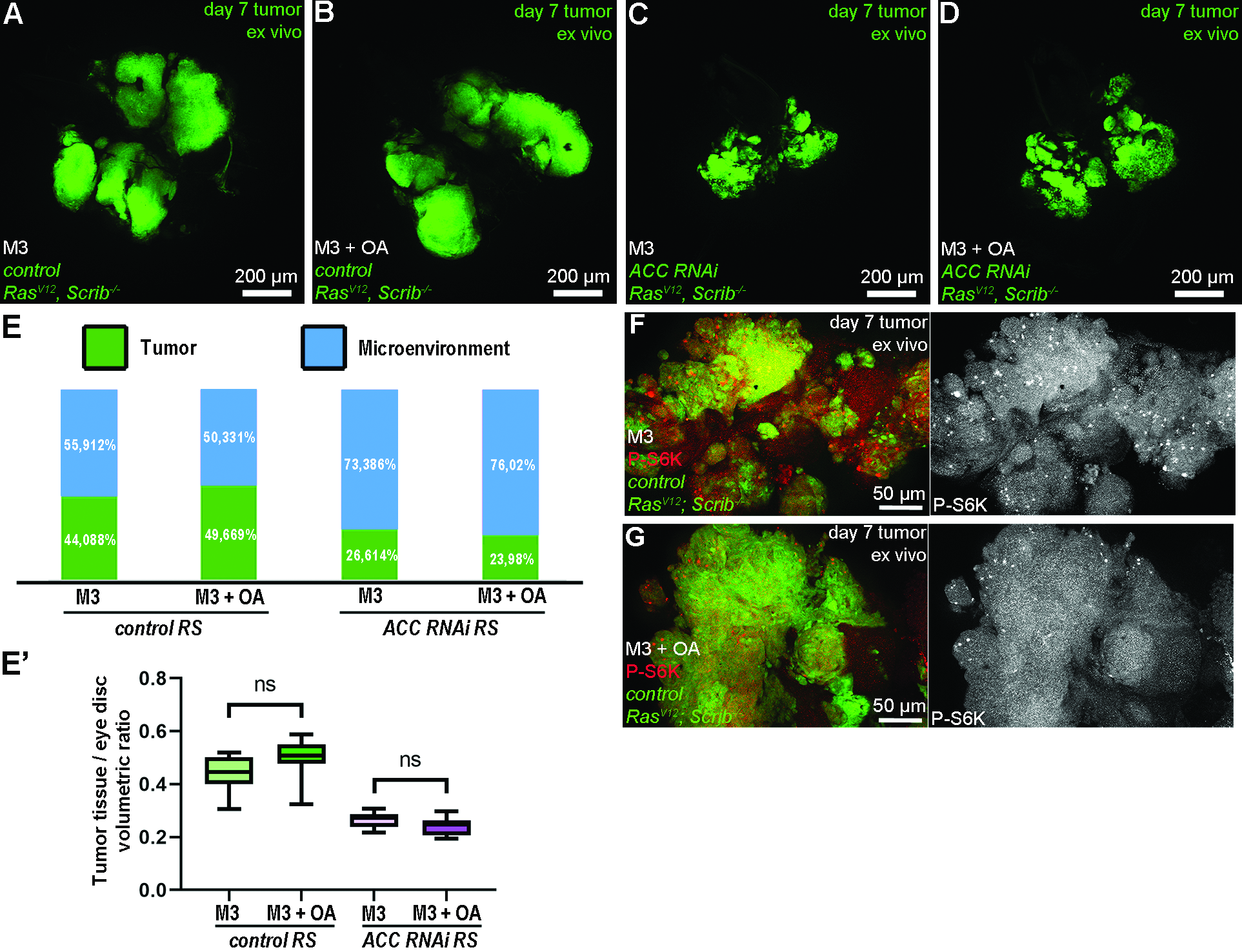

Supplement: Supplementary file 7 — Supplementary Fig. S6 [file 41419_2026_8738_MOESM7_ESM.tif]
