# Supplementary material for: Fatty acid synthesis supports tumor progression through facilitating the activity of TORC1 signaling
Source: Cell Death Dis. 2026 Apr 10;17(1):468. doi: 10.1038/s41419-026-08738-6 (PMC13181055; doi:10.1038/s41419-026-08738-6)
Supplement: Supplementary file 11 — Supplementary Table S4 [file 41419_2026_8738_MOESM11_ESM.pdf]

Lipid Class	Internal standard (IS)	Ion format	Conc of infusion (fmol/μL)
PC, LPC	PC(15:0/18:1-d7)	[M+H]+	480
PE, LPE	PE(15:0/18:1-d7)	[M−H]-	170
PI, LPI	PI(15:0/18:1-d7)	[M−H]-	119
PS	PS(15:0/18:1-d7)	[M−H]-	129
PG	PG(15:0/18:1-d7)	[M−H]-	26
PA	PA(15:0/18:1-d7)	[M−H]-	29
CL	CL(tetra14:1)	$[M-2H]^{2-}$	16
Cer	Cer(t18:1/16:0)	[M+Cl]−	36
HexCer	GluCer(d18:1/12:0)	[M+Cl]−	31
CerPE	CerPE(29:1:2)	[M−H]-	340
DG	DG(15:0/18:1-d7)	[M+NH4]+	34
TG	TG(15:0/18:1-d7/15:0)	[M+NH4]+	124
EE	Chold7E(16:1)	[M+NH4]+	153
