# Supplementary material for: Fatty acid synthesis supports tumor progression through facilitating the activity of TORC1 signaling
Source: Cell Death Dis. 2026 Apr 10;17(1):468. doi: 10.1038/s41419-026-08738-6 (PMC13181055; doi:10.1038/s41419-026-08738-6)
Supplement: Supplementary file 13 — Original Data [file 41419_2026_8738_MOESM13_ESM.pdf]

Original Data

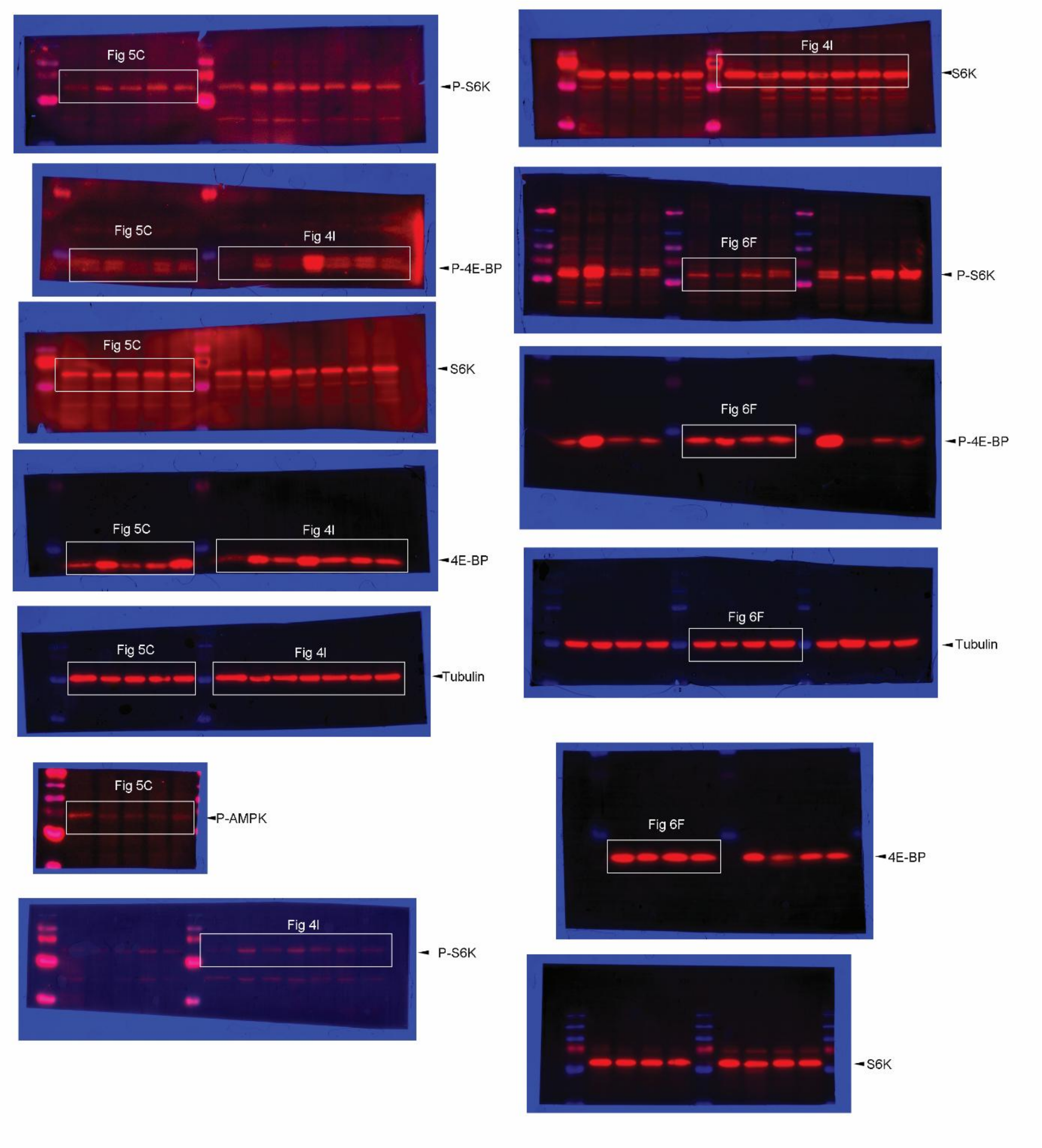

Uncropped membranes for western blot experiments shown on Fig. 4I, Fig. 5C and Fig6F. The cropped and edited parts of the membranes are encircled in white.
